# Polyelectrolyte Gels: Fundamentals, Fabrication and Applications

**DOI:** 10.3390/gels7030148

**Published:** 2021-09-18

**Authors:** Nisal Wanasingha, Pramod Dorishetty, Naba K. Dutta, Namita Roy Choudhury

**Affiliations:** School of Engineering, STEM College, RMIT University, Melbourne, VIC 3000, Australia; s3487229@student.rmit.edu.au (N.W.); s3708732@student.rmit.edu.au (P.D.)

**Keywords:** polyelectrolytes, gels, stimuli-responsive, multilayer gels

## Abstract

Polyelectrolyte gels are an important class of polymer gels and a versatile platform with charged polymer networks with ionisable groups. They have drawn significant recent attention as a class of smart material and have demonstrated potential for a variety of applications. This review begins with the fundamentals of polyelectrolyte gels, which encompass various classifications (i.e., origin, charge, shape) and crucial aspects (ionic conductivity and stimuli responsiveness). It further centralises recent developments of polyelectrolyte gels, emphasising their synthesis, structure–property relationships and responsive properties. Sequentially, this review demonstrates how polyelectrolyte gels’ flourishing properties create attractiveness to a range of applications including tissue engineering, drug delivery, actuators and bioelectronics. Finally, the review outlines the indisputable appeal, further improvements and emerging trends in polyelectrolyte gels.

## 1. Introduction

Polymeric gels are flexible semisolid structures holding large quantity of fluid in the interstitial spaces of physically or chemically crosslinked polymeric networks [1]. These gels exhibit both elastic and viscous properties arising from the polymer networks and fluid, respectively. On the other hand, polyelectrolyte (PE) gels are polymer gel networks with charged groups or ionisable moieties stabilised by several interactions, including electrostatic interaction, van der Waals forces, ionic interactions, hydrogen bonding and chemical crosslinking [2]. They have the capability to hold a large amount (~2000 times the polymer weight) of water/solvent in the polymer network without dissolving [3]. Unlike conventional gels, PE gels offer additional complexities due to the dynamic nature of the charged groups and their density differences present in the polymeric backbone [4]. PE gels are also found in mammalian tissues such as cartilage, where its elasticity arises from the PE complexes stabilised by proteins. For example, the PE complex formed between oppositely charged aggrecan and hyaluronic acid (HA) is interspersed in the collagen matrix, which mimics the extracellular matrix (ECM). The bottlebrush architecture of aggrecan helps in providing characteristics required to resist deswelling during compressive loads [5]. PE gels can also be responsive to external stimuli such as pH, ionic strength, temperature, electricity and light, making them an attractive candidate for both biomedical and industrial applications [6].

In general, PE gels are produced by several approaches, including combining the oppositely charged polymers, stabilisation of PEs with oppositely charged surfactants and crosslinking the PE polymer by activating the reactive groups [2,7]. The selection of PE gels for biological applications is attractive due to their favourable intrinsic and extrinsic characteristics. For instance, researchers are attracted to cationic polymers such as gelatin and chitosan (CHT) for drug delivery applications, not only due to their biocompatibility and strong interactions with proteins and DNA but also for their ability to bind to negatively charged surfaces. In turn, there is a significant improvement in interactions with the targeted cell membrane, thereby presenting itself as an effective transfection agent [8]. In comparison, there is a higher inclination to use anionic polymers for bioadhesive applications with carboxylic groups that can interact with the free amine groups found in tissue, thereby providing an effective and durable adhesive bond [9]. Unlike conventional crosslinked gels, PE gels have additional complexities because of the interplay between charged ions and other physical factors. Despite these complications, PE gels have flourished by incorporating numerous materials with various stimuli-responsive behaviours, resulting in attractive properties. In turn, PE gels have presented astounding potential, being implemented into a vast array of applications such as drug delivery [10,11], tissue engineering [12,13], electronics [14,15] and actuators [16].

**Figure 1:**
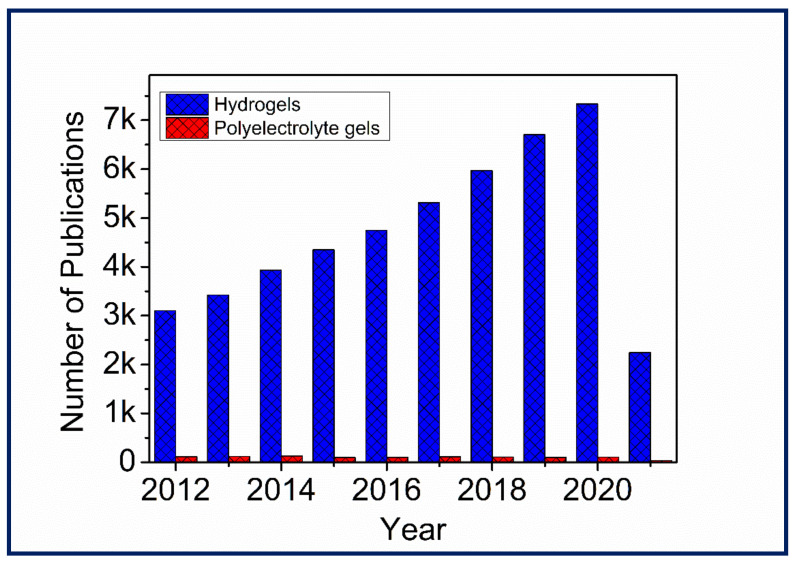
Number of publications over the past ten years by searching “Hydrogels” and “Polyelectrolyte gels” in Scopus on 27 March 2021.

**Figure 2:**
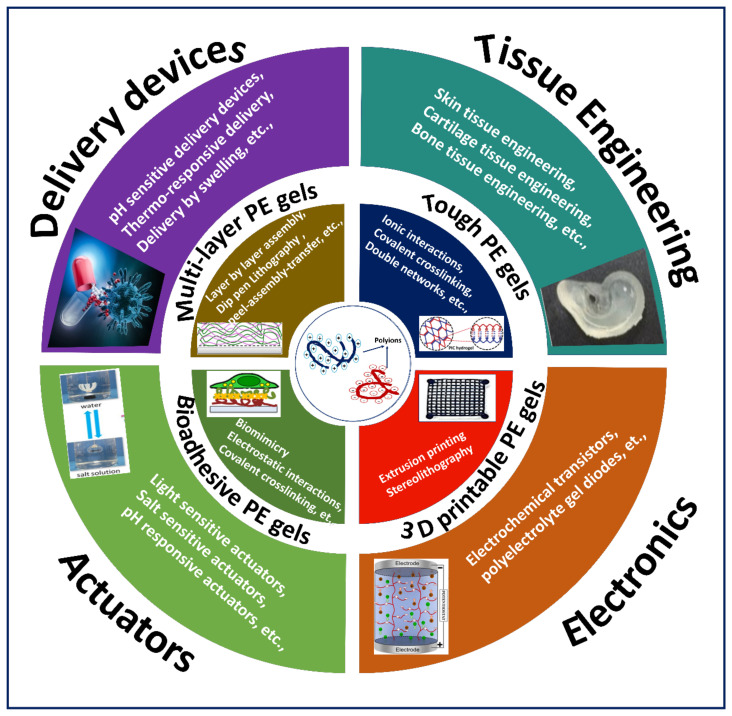
Different types of PE gels and applications.

Although PE gels showed excellent stimuli-responsive properties required for various applications, the number of studies on PE gels in the last decade was significantly lower when compared to the increasing studies on hydrogels (Figure 1). However, there have been a few reviews that discuss PE gels produced by interactions and crosslinking as a subsection in PE complex formations [6,17,18,19]. Moreover, PE gel reviews focusing on electrical properties, diffusion of proteins in PE gels, negatively charged PE gels and PE gels for cell encapsulation were published over a decade ago [2,20,21]. To our knowledge, there has been no article on compiling the recent literature on PE gels produced by exploiting ionic interactions and crosslinking mechanisms with advanced fabrication techniques. Hence, this review is intended to guide the reader through the recent literature on the fabrication of PE gels, focusing on highlighting the exploitation of ionic interactions and crosslinking mechanisms coupled with advanced fabrication approaches to construct various types of demanding gels for different applications (Figure 2).

## 2. Fundamentals of PE Gels

The concept of PE gels revolves around judiciously using both anionic and cationic constituents to form a gel. Depending on various phenomena of the PE chosen, PEs can fall under different classifications, which are the root source of properties they attain. Furthermore, these properties influence different aspects of PE gels, such as stimuli-responsive behaviour and ionic and electrostatic interactions. In turn, the combination of the classifications and aspects obtained from the PE influences the overall properties and characteristics exuded by the gel.

### 2.1. Classification of PE Gels

**Figure 3:**
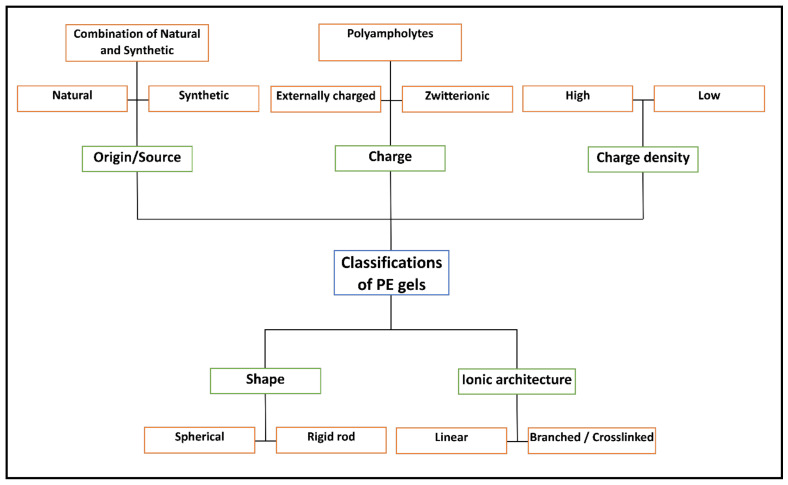
Classifications of PE gels.

PE gels are predominantly classified based on their origin, commonly developed by using natural, synthetic or, in some cases, a combination of both for PEs to utilise the properties obtained from both sources. However, with a multitude of PEs to select from, further classifications are observed, such as charge, charge density, shape and ionic sites’ location, as presented in Figure 3.

PE gels that are classified as natural are typically based on materials obtained from the natural world. The natural biopolymers that are commonly observed as major constituents in PE gels include polymers such as CHT, HA and heparin (HEP) [22,23]. These glycosaminoglycans (GAGs) are naturally found in the ECM at various locations, particularly the connective, neural and epithelium tissue. Moreover, they exhibit attractive properties such as non-toxicity, cytocompatibility and aiding attachment and cell growth [24]. Thus, these features stand out for biological applications with their ability to mimic and present the biochemical/mechanical properties of the ECM. Other common natural polyelectrolytes are pectin (polygalacturonic acid), alginate (alginic acid), carboxymethyl cellulose and polypeptides. However, gels formed through the means of natural PEs generally present weak mechanical properties that hinder their full potential in many application [25]. Therefore, PE gels need to be produced in a manner that does not compromise the native properties of the bioactive polymers whilst also conforming to the application’s needs. The fabrication of PE gels from natural materials by various approaches is discussed in the section exploring the fabrication of different PE gels.

Analogous to natural PEs, synthetically derived PEs are widely utilised for exhibiting wide tuneability in physical and chemical properties. The best known synthetic polyelectrolyte gels are those used in super absorbents and ion exchangers. Polyacrylic acid, polystyrene sulfonate, polyallylamine, carboxymethyl cellulose and related salts are examples of typical synthetic polyelectrolytes. Typically, a distinction is noted between natural and synthetic PEs, with the former having rigid backbones and longer persistence lengths—representing rigid backbones and a higher bending stiffness. In contrast, the latter are mainly derived from vinyl monomers, where their backbones are less rigid and display higher flexibility throughout the carbon chain, with shorter persistence lengths. Therefore, synthetic PEs present greater effective compensation of charges, as natural PEs are further weakened by the lower charge densities they obtain [26,27]. Despite the advantages seen over natural PEs and the wider use in development, synthetic PEs have setbacks associated with exhibiting a poorer biocompatibility profile and biological activity than natural PEs. Both natural and synthetic PEs have also been utilised to form interactions with proteins to further expand the potential of PEs for biomedical applications. The surface of proteins typically consists of positive and negative charges, where they usually bear a net negative charge (dependent on the pH of the environment). In this regard, when PEs interact with a protein, counterion release force is observed. Briefly, when a highly charged PE chain interacts with a protein, it becomes multivalent counterions of the PE. In turn, the counterion release force becomes the major driving force of interaction between the protein and PE, further displaying a gain in entropy [28,29].

Another classification of PE gels is associated with the integral charge formation. Typically, charges are classified upon counterions from two oppositely charged PEs encountering each other through external interaction, resulting in a final charge being obtained. However, another type of charge classified from PE gels that are less frequently explored is PEs that are composed of subunits of oppositely charged polymers producing internal interactions to form a charge. Both types of classifications present wide tuneability in stimuli responsiveness, mechanical properties, etc.; however, the final properties displayed can differ according to the manipulation applied. For instance, polyampholytes typically present enhanced swelling and dissolution due to screened interactions compared to PEs of the same subunits that prepare the gel in the conventional way [30,31]. In contrast, zwitterionic PE gels are recognised as having a charge density that is neutrally charged. These PE gels also demonstrate multifunctional tuneable properties but have been seen to stand out more than the former two categories in specific properties. For example, zwitterionic PE gels exhibit significantly greater conduciveness than pure PE gels [32,33].

Other major classifications in PEs are observed as weak and strong, which are determined by their capacity to dissociate in solution. In strong PEs, the polymeric chains are completely ionised and chairs are in an extended conformation [34]. On the other hand, weak PEs possess dynamic conformations, which can interchange between coiled and rigid conformations with a slight change in pH [35]. In relation to PEs’ classification based on their charge, another classification is the charge density and ionic strength of the PEs selected, which can highly influence the gel’s application. Evidently, the relationship between ionic strength and charge density influences numerous factors stemming from the formation of the gel, thereby affecting other behaviours such as release kinetics, adsorption/desorption and mechanical properties [36,37]. For instance, PEs with high charge densities such as poly(diallyldimethylammonium chloride) (PDADMAC) and poly(styrenesulfonate) (PSS) typically exhibit intrinsic links that transform into extrinsic linkages when the ionic strength rises [38].

Regardless of the source, the extent to which charges present themselves and influence the properties of the gel can be classified by their shape and ionic architecture. PEs that are classified by shape are commonly labelled as either spherical or rigid rods. Spherical PE gels are commonly recognised as globular proteins, whereas rigid rod PEs attain their structure through the linear configuration of repeating units in their backbone such as poly(2,2′-disulfonyl-4,4′-benzidine terephthalamide) [39,40]. In parallel, PE gels are classified based on the ionic architecture of the PE, which is categorised based on the position of the ionic sites. The architectures are classified as either linear or branched/crosslinked. The former can be further distinguished depending on where the ions are located on the backbone (integral or pendant) [41,42].

### 2.2. Important Aspects in PE Gels

#### 2.2.1. Conductivity

**Figure 4:**
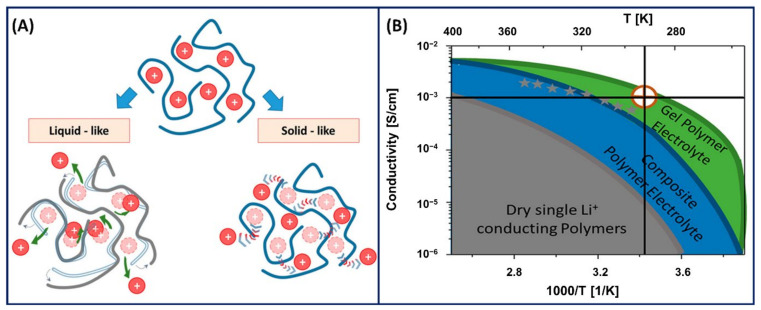
(**A**) Schematic presentation of two possible mechanisms of ion transport in polymers: the liquid-like mechanism (lower left) requires the motion of the polymer segment and depends on the rate of segmental relaxation, whereas the solid-like mechanism (lower right) is based on ion jumps over an energy barrier in the frozen (on the time scale of ion jumps) polymer matrix. (**B**) Schematic presentation of developing various types of polymer electrolytes, where the cross in the circle marks the value for conductivity at the ambient temperature required for practical applications. The stars represent single dry Li^+^ conducting block polymers reported in literature Reprinted (adapted) with permission from [44]. Copyright (2020) American Chemical Society.

The conductivity or migration of ions is an important feature of PE gels that can be exploited in developing flexible and stretchable electronic devices. The electrical conductivity in PE gels inherently depends on the ionic conductivity, where ions carry electrical currents. However, the performance of the devices is determined by the migration ability of the ions when subjected to an electric field. Although an electric field triggers ionic migration or conductivity, the driving force at natural conditions is by the concentration gradients, creating ion diffusion. The ionic conductivity (*σ*) in polymer electrolytes is estimated by the classical Nernst–Einstein (NE) equation, Equation (1), which depends on the charge, concentration, temperature and diffusion [43].

(1) $$\sigma =\frac{1}{kT}\;\underset{i}{\sum }{n_{i}q_{i}}^{2}D_{i}$$

where *n_i_* is the concentration, *q_i_* is the charge of the free ions contributing to conductivity, and *D_i_* is the diffusion coefficient. In general, the ionic conductivity or transport in polyelectrolytes is due to the segmental relaxation by either liquid-state or solid-state mechanism (Figure 4A) [44]. In the liquid-state mechanism, the diffusion of ions is governed by the local friction generated by the polymer viscosity and structural relaxation time [45]. Comparatively, in the solid-state mechanism, the ionic conductivity arises from the ions jumping over an energy barrier controlled by electrostatic interactions and elastic forces [46]. In addition to a liquid-state or solid-state ion transfer mechanism, the ionic conductivity significantly depends on the ion–ion correlations. The experimental ionic conductivity measured for various polymer electrolytes is significantly lower than the ionic conductivity measured using Equation (1) because of the additional ion–ion correlations coming into the picture [47]. The decrease in ionic conductivity in polyelectrolyte polymers is due to the participation of cation–anion pairs only in ion diffusion and not the charge transport and conductivity [48]. In addition, there is a possibility of multiple anions taking a cation in the opposite direction to the electric field, which significantly reduces the overall ionic conductivity. Therefore, the ion–ion correlations are confined to dilute and semi-dilute polyelectrolytes. The ion–ion correlations are difficult to interpret for highly concentrated polyelectrolytes or in PE gels because of the uncertainty in the dynamics of the polyions surrounded by the counterions.

Polyelectrolytes with high concentrations present the complexity of ion–ion correlations and decreased ionic conductivity (estimated theoretically), where PE gel networks capable of holding large amounts of solvents or liquid plasticisers show excellent ionic conductivity at room temperature (Figure 4B) [44]. The presence of a solvent accelerates the ion and polymer segmental dynamics and provides an alternative ion transport through the liquid medium, resulting in an increased ionic conductivity in PE gels. However, the ionic conductivity in PE gels is highly complex and less understood because it depends on other factors such as the concentration, aggregation, charge density, network structure or crosslink density [49,50].

#### 2.2.2. Stimuli Responsiveness

Stimuli responsiveness is an integral characteristic of PE gels because of the dynamic nature of the polymer networks and the ability to respond to a range of stimuli [51]. Responsiveness is typically classified as either chemical (pH, ionic strength, etc.) or physical (temperature, light, etc.). Properties such as the shape, elasticity and volume of the gels can be manipulated depending on the polyelectrolyte’s responsiveness. As the next part of this review covers different types of PE gel fabrication and applications by highlighting the responsiveness of PEs to different stimuli, the following section will briefly cover the impact of common stimuli of polyelectrolyte gels.

The most common physical response used to form and control polyelectrolyte gels is temperature. Temperature-responsive polyelectrolyte gels are observed when a polyelectrolyte chain undergoes conformational changes depending on the temperature, thereby influencing the interactions that occur. Complex polyelectrolyte coacervate gels were formed with increasing temperature of a mixture of poly(*N*-isopropylacrylamide) (PNIPAM) content and poly(2-(dimethylamino) ethyl methacrylate) (PDMAEMA). With an increase in temperature, the polyelectrolytes’ responsiveness resulted in enhanced gelation and adhesion strength, where the network could retain hydrophobic domains and enabled injection of the gel through the increase in viscosity [52]. Similarly, deswelling of the gel can also occur with polyelectrolytes when they attain critical solution temperatures [53].

Light-responsive polyelectrolyte gels have been developed with the ability to display light-triggered swelling and shrinking. These gels are highly beneficial for various applications such as drug delivery, tissue engineering and soft actuators, where light exposure triggers a fast, responsive polyelectrolyte. Nakajima et al. [54] developed a tough polyelectrolyte gel by exploiting the light-responsive polymerisation technique. Briefly, the first network is formed via UV light, and it is further immersed in the second network of reactants to swell again and photopolymerised for a final time to form a strong double-network gel.

Materials that display chemical changes are commonly observed in gels, and the same can be applied to polyelectrolyte gels. Ionically responsive gels display networks that constitute polyelectrolytes. These polyelectrolyte gels form through swelling which occurs due to osmotic pressure gradients, dependent on the environmental conditions [55]. In response to the environmental conditions that entail pH or ionic concentrations, the polyelectrolyte gel exhibits conformation changes kindred to physical responsiveness such as strength, permeability and elasticity.

#### 2.2.3. Crosslink Density of PE Gels

The crosslinking density (S) in elastic polymeric gels can be defined in different ways, which mainly revolve around the concept of equilibrium swelling and elasticity. The former can be determined using Equation (2), which is based on the Flory–Rehner theory [56], whereas the latter can be estimated from the rubber elasticity theory using Equation (3) [57].

(2) $$S=\frac{\rho }{M_{c}}=\frac{-\ln\left(1-V_{1,s}\right)+V_{1,s}+\chi V_{1,s}}{V_{2}\left(V_{1,s}-\frac{1}{2}V_{1,s}\right)}$$

(3) $$S=\frac{E}{3RT}=\frac{G}{RT}=\frac{\rho }{M_{c}}$$

where *ρ* is the density of the gel, *M_c_* is the molecular weight between two adjacent crosslinks, *V*_1,*s*_ is the polymer volume fraction in the swollen state, *V*_2_ is the molar volume of the solvent, *χ* is the Flory–Huggins interaction parameter between the polymer and solvent, *E* represents the tensile elastic modulus (Pa), *G* is the storage modulus (Pa), *R* is the universal gas constant (8.314 J mol^−1^ K^−1^), and *T* is the absolute temperature (298 K).

Comparatively, the crosslinking density of PE gels involves additional complexities arising from the electrostatic interactions, ionic exchanges, etc. However, to understand the crosslinking density in PE gels, there needs to be further understanding into competing thermodynamic forces that fall under Helmholtz free energy (Δ*F*), Equation (4)), which has been thoroughly reviewed [58]. Briefly, Δ*F* incorporates additional factors required to determine the crosslink density, such as the dependence on the volume fraction (φ), degree of ionisation (α), salt concentration and Flory–Huggins χ parameter.

(4) $$\Delta F=\Delta F_{mean\;field}+\Delta F_{fluctuations}$$

The free energy of the mean field (Δ*F_mean field_*) consists of mixing, electrostatic interactions, elasticity and the Donnan equilibrium for electrolytes, whereas the free energy fluctuations (Δ*F_fluctuations_*) relate to fluctuations in chain connectivity such as conformation and polymer concentrations. In turn, the sum of the contribution from the terms is determined by the osmotic pressure (Π) arising from Δ*F*, thus resulting in Equation (5).

(5) $$\Pi =\Pi_{mix}+\Pi_{electrostatic}+\Pi_{elastic}+\Pi_{Donnan}+\Pi_{fluctuations}$$

Of the terms that summate to the osmotic pressure, Π*_mix_* is derived from the Flory–Huggins theory of mixing observed in Equation (6):

(6) $$\frac{\Pi_{mix}v_{1}}{kT}=-\ln\left(1-\phi \right)-\phi -\chi \phi^{2}$$

where *kT* is the Boltzmann constant, and *v*_1_ is the volume of the solvent.

The second term, Π*_electrostatic_*, corresponds to all the electrostatic interaction energy found among the gel network at a given salt concentration, which can be simplified down to Equation (7).

(7) $$\frac{\Pi_{elec}v_{1}}{kT}=0$$

The contribution of Π*_elasticity_* is derived from the rubber elasticity theory, which accounts for the isotropic swelling of the PE gel seen in Equation (8):

(8) $$\frac{\Pi_{elastic}v_{1}}{kT}=-\frac{1}{N}\left(\phi_{0}^{2/3}\phi_{0}^{1/3}-\frac{\phi }{2}\right)$$

where *N* is the number of segments within a chain. Therefore, Equation (8) confirms how Π*_elasticity_* is negative, thereby working against the swelling property.

Π*_Donnan_* is determined from the osmotic pressure of equilibrated electrolytes (Π*_ions_*_)_ that exchange in and out of the gel until charge neutrality and a constant chemical potential, given by Equation (9).

(9) $$\frac{\Pi_{ion}v_{1}}{kT}=\sqrt{{ɑ}^{2}z_{p}^{2}\phi^{2}+4v_{1}^{2}c_{s}^{2}}-2v_{1}c_{s}$$

where *z_p_* is the valency of the segment, and *c_s_* is the salt concentration.

The fluctuation in the gel can also influence the total osmotic pressure free energy, but predominantly when it is near the critical point of the gel. Thus, the term is omitted from the final summation of the osmotic pressure of the gel in this example that combines Equations (6)–(9), resulting in the swelling equilibrium of PE gels given by Equation (10).

(10) $$\frac{\Pi v_{1}}{kT}=-\ln\left(1-\phi \right)-\phi -\chi \phi^{2}+\sqrt{{ɑ}^{2}z_{p}^{2}\phi^{2}+4v_{1}^{2}c_{s}^{2}}-2v_{1}c_{s}-\frac{1}{N}\left(\phi_{0}^{\frac{2}{3}}\phi_{0}^{\frac{1}{3}}-\frac{\phi }{2}\right)$$

Finally, we can understand that *N* is proportional to the swelling ratio. Thus, an increase in *N* will result in a decrease in the crosslinking density, where the relationship can be understood by Equation (11).

(11) $$S=\frac{\frac{1}{N}}{\phi_{0}^{2}}$$

As previously mentioned, the theoretical crosslinking estimated in PE gels is primarily based on the competing thermodynamic conditions. However, considering the complexity of PE gel systems, the inhomogeneity in these gel networks is poorly understood and characterised. To address the inhomogeneities found in the crosslinked network, small-angle neutron scattering (SANS) is a powerful characterisation technique that determines the structural information of materials across smaller length scales (nano- to microscale) [59]. For instance, the increase in the crosslinking density in a carboxymethylated thiolated hyaluronan (CMHA-S) and polyethylene glycol diacrylate (PEGDA) gel resulted in a difference in the static structural difference due to the inhomogeneities present in the crosslinks, which was captured by the power law regime of the SANS intensity profile [60]. Despite SANS providing structural inhomogeneities of gels, the relationship between SANS structural parameters and thermodynamic relations is still in the early stages.

## 3. Fabrication of Different Types of PE Gels

PE gels feature several advantages such as excellent tuneability, biocompatibility and stimuli-responsive properties. PE gels further offer the tuneability of cellular activities such as adhesion and proliferation by exploiting the cell–gel interactions [61]. Therefore, to exploit the excellent characteristics offered by PE gels, different types of demanding PE gels (Table 1 in Section 3.4) are fabricated for diverse applications.

### 3.1. Multilayered PE Gels

Multilayered systems are an essential class of functional materials that allow a wide range of tuneability arising from their unique assembly, enabling the gel to be applied for various applications. However, multilayer functional materials’ efficiency depends on both intrinsic material properties and the fabrication approach. Of the assortment of technologies/techniques available, layer-by-layer (LBL) assembly is a dominant technique known for its high reproducibility, tuneability, efficiency and ease of fabrication. This prevalent technique was first introduced for colloidal particles in the mid-1960s [10], where it was only repurposed for PEs roughly two and a half decades later [62,63].

As LBL assembly requires a synergy/interaction between layers, PE gels have been exploited in this manner. They commonly exhibit ionic and electrostatic interactions to create a strong network structure, presenting self-assembly in an LBL construction. The self-assembly of the layers is highly dependent on the intrinsic properties of the PE (composition, concentration, etc.) in addition to the external conditions (pH, ionic strength, etc.) [64,65]. As a new means of LBL assembly, understanding the influence and control of multilayers allows the development of advanced PE multilayers (PEMs) [66].

A prominent feature influenced by numerous properties of PEMs is the growth regimes observed during assembly, which are categorised as either linear or exponential. The linear regime follows a constant growth pattern, where the thickness correlates with the number of layers present. In contrast, exponential growth is associated with polymer dynamics. For example, when the adsorbed amount of PEs within the build-up of multilayers is proportional to the multilayer thickness, exponential growth is attained [67,68]. Therefore, it can be inferred that exponential growth of PEMs can only be achieved if one of the PEs present is capable of diffusing through the multilayers in the complex [69].

**Figure 5:**
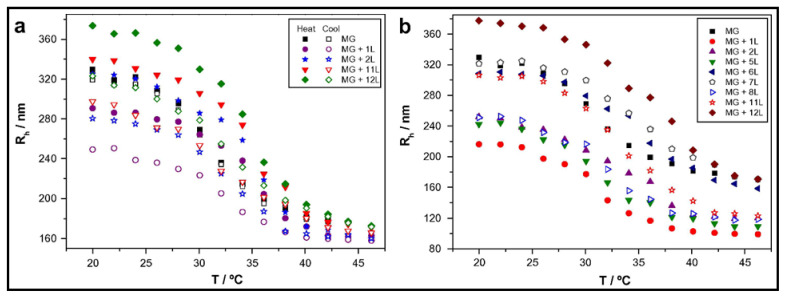
Change in *R*_h_ of PNIPAM-co-MAA nanogels coated with either (**a**) PLL/PGA or (**b**) CHT/DS. Reproduced from [73].

Temperature variations have been shown to influence the growth of PEMs as well as additional properties post-assembly. The deposition temperature of each layer reflects on the internal interactions that occur within the complex. For instance, in one study, a positive linear correlation between an increase in thickness and temperature to a predetermined set point was found [70]. As gels require water content to be present, the effect of temperature is limited in the bilayers’ thickness compared to films. Specifically, exponential to linear growth in multilayers has been observed with increased temperature due to the limited polymer diffusion once the transition point is met [71]. However, the influence of temperature on PEMs has been shown for surface modification of thermoresponsive microgels, leading to intriguing effects [53,72]. Wong and Richtering [53] coated PEMs (PDADMAC/PSS) onto a PNIPAM acrylic acid core and PNIPAM shell microgel, which displayed a broader lower critical solution temperature (LCST) (~36 °C) in comparison to a PNIPAM microgel with a sharp LCST at ~32 °C. The thermoresponsive nature led to both architectural and size changes in the resulting gel [53]. In an analogous study, the hydrodynamic radius (*R*_h_) of a PNIPAM-co-methacrylic acid (PNIPAM-co-MAA) nanogel coated with either poly(L-lysine) (PLL)/poly(L-glutamic acid) (PGA) (Figure 5a) or CHT/dextran sulphate (DS) (Figure 5b) multilayers was compared with increasing temperature [73]. As seen in Figure 5, after adsorption of the first cationic layer, there is a decrease in the *R*_h_ of the gel; however, the thermoresponsive behaviour is retained. The second layer also retains the thermoresponsive behaviour; however, it displays an increase in size. Thus, an “odd–even” effect is found depending on the charge of the outermost layer. Comparing both microgels, upon the approach of the anionic PEs (PGA or DS), the opposite charges need to distribute evenly between the gel and the PE. Hence, the *R*_h_ decreases, as the pulling attraction between the gel and cation is decreased.

The salt concentration and ionic strength are also noted as major parameters that influence the growth of PEMs and the resulting structure and stability. Initially, PEMs were developed using strong PEs that are fully charged and independent across a range of pH, where the thickness of the multilayers was manipulated by the addition of salt [74]. Despite the advantages presented, this strategy was limited to the extent that strong PEs with high molecular weights, such as PSS and PDADMAC, have low solubility in a solution that presents high ionic strengths. For instance, the addition of a salt concentration hinders the properties of the gel, where the PEM composition would be dampened. Thus, to overcome this setback, weak PEs such as PLL and PGA were found as an alternative approach. These weaker PEs showed greater responsiveness in assembly over stronger PEs, mainly due to the latter forming strong ionic crosslinks that limit their mobility [75]. One example is a change in structure, where PLL was shown to alter with increasing temperature at a low salt concentration, where it changes to a folded β-sheet structure from an unordered random coil state and retains its structure once the solution is cooled. The addition of salt is further seen to result in swelling of the multilayer, enabling each layer to bind to each other, promoting localised dissociation and greater conformational dynamics. In turn, the PEM gel system has a greater mobility of the polymeric chains, enabling hysteresis and displaying potential for the storage and release of biomolecules [73].

**Figure 6:**
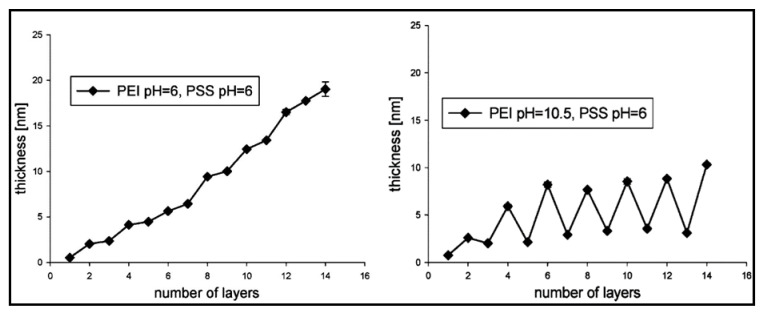
Influence of changing the pH of a weak PE, PEI, on the final thickness of the PEM. Reprinted (adapted) with permission from [78]. Copyright (2009) American Chemical Society.

Functionalisation of PEM gels based on pH responsiveness has widened applications, particularly in the biomedical field, such as implants, drug delivery, microfluidics and biosensing [76,77]. In many cases, in conjunction with the ionic strength of PE solutions, the final stability of PEM gels is highly dependent on pH. Furthermore, analogous to the influences that strong and weak PEs have shown for other parameters, they behave differently under various pH conditions. Elzabieciak et al. [78] exemplified this matter, where they showed a distinct difference in the multilayer thickness when polyethyleneimine (PEI) was under pH 6 and pH 10.5; the latter resulted in a gradual oscillating increase, in comparison to a linear increase with pH 6. The stronger PE (PSS) was not varied in pH due to previous studies deeming it not pH-dependent. Furthermore, Figure 6 illustrates that when the total number of layers is odd (ending in a PEI layer), it would result in a drop in thickness due to the weaker PE being produced at pH 10.5. The trend obtained could be supported by understanding the formation of weakly bound complexes with greater permeability that can be partially removed from each absorption step on the surface, resulting in a thinner thickness than a strongly charged polycation (at pH 6). The hydrodynamic radius (*D*_h_) of microspheres composed of PEMs has also demonstrated pH sensitivity. Specifically, the *D*_h_ of PNIPAM grafted onto multilayers of CHT and alginate (ALG) had a significant decrease (~930 nm) with an increase in pH (2 to 10). The trend observed for the *D*_h_ was based upon the decline in the ionisation of the amine groups of CHT after crossing a pH value of 6, resulting in greater electrostatic interactions between both cationic and anionic PEs [79]. Similarly, responsiveness to pH is exploited for the swelling capacity of PEMs, which aids in release applications such as drug delivery and tissue culture [80].

To endow further responsiveness to PEM gels, particularly for biological applications, crosslinking plays a role in influencing various properties of the gel by altering the degree of crosslinking and conformational changes occurring from internal crosslinks between polymeric chains [11,81,82]. Decreasing the degree of crosslinking improves PEM gels’ swelling properties, where the crosslinked network would be less compact, and polymer chains become less rigid [11]. Thus, low degrees of crosslinking allow PEM films to function as hydrogels [83]. In turn, attaining these properties would aid in applications such as drug encapsulation, where the loading capacity can further increase [11]. In other studies, frequently used crosslinking techniques such as incorporating N-hydroxysulphosuccinimide (NHS) and 1-ethyl-3-(3-dimethylaminopropyl)carbodiimide (EDC) into PEM films have further shown modifications in surface roughness, wettability and stiffness, which are applicable to gels [84]. Taking stiffness as an example, multilayered structures were shown to increase up to 10-fold due to the crosslinked network obtained [85]. Analogously, crosslinked PEMs have largely been shown to influence cell adhesion and proliferation [81,86]. Compared to PEM gels presenting structures with low rigidity, Yamanlar et al. [87] suggested that enforcing rigidity in the structure allows adhesion to occur, which is possible through crosslinked PEM films. Moreover, introducing films with HA hydrogels results in changes in the gel’s physicochemical properties, further rendering it to become cell-adhesive.

In conjunction with properties that influence performance, the LBL assembly method chosen further impacts the properties of the PEM gel. In general, there are three conventional techniques used for LBL assembly. These entail spin coating, dip coating and spray coating. Despite having slight similarity to conventional methods, advanced PEMs enable further diversity in application, presenting a range of trajectories unmatchable with conventional methods to fabricate multilayers for future designs. Some examples include creating film patterning, which presents difficulties in assembling at nanoscales, expansion in biological assemblies (particularly on soft, biocompatible substrates) and control of LBL assembly at both smaller and larger scales than the conventional method [88,89].

**Figure 7:**
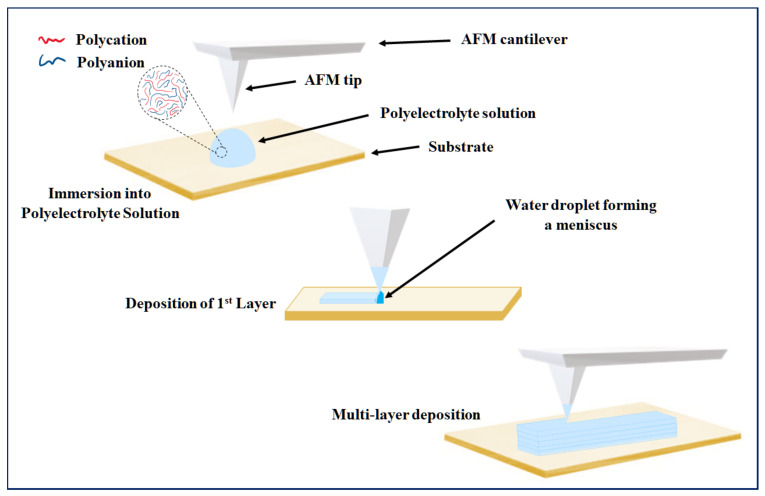
Schematic of DPN technique used to form PEMs.

Advanced PEM assembly has been achieved through interdisciplinary approaches, where variations in nanofabrication methods associated with lithography are widely used, which enable layered patterning in a controlled manner [90]. Of the various lithographic methods, dip pen nanolithography (DPN) is well known and widely utilised, where several derivations of the technique have been explored [91,92] and are still gaining interest for use in LBL assembly. The technique incorporates the use of an atomic force microscope (AFM) cantilever tip, where it is used as a writing device to deliver the relevant chemical as an ink in a predetermined pattern, as shown in Figure 7. Despite the slow speed in patterning compared to other techniques, the attractiveness of DPN is highlighted in its ability to create arbitrary patterns, ranging in sizes and shapes on a single substrate, allowing accurate, high-spatial resolution construction of multilayers that form interactions at a nanoscale [93].

**Figure 8:**
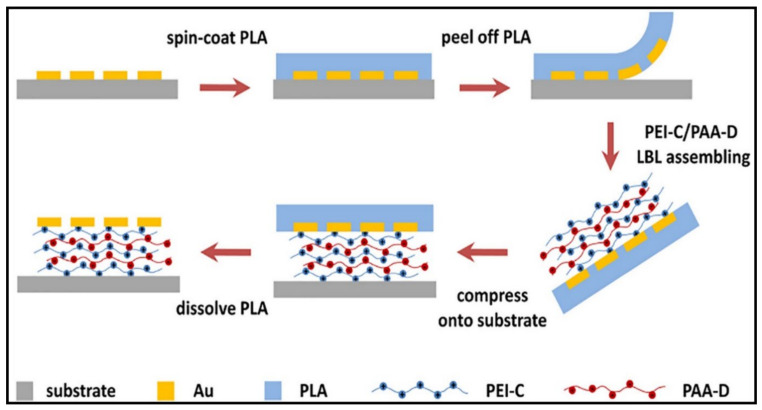
Construction on PEMs through the PAT procedure. Reproduced from [95].

A vastly uncommon yet advanced technique used to assemble multilayers is the peel-assembly-transfer (PAT) method. The strategy behind this technique revolves around introducing polymers between plasmonic microstructures and substrates, allowing the various secondary interactions to form a strong network [94]. Jiao et al. [95] utilised the LBL technique to develop a mussel-inspired PE using the PAT procedure to incorporate polyelectrolytic monomers (polyetherimide and poly(acrylic acid) (PAA)) into a multilayer adhesive system, as shown in Figure 8. A film is peeled off from a substrate, followed by an LBL assembly of PEMs, which are then compressed onto a substrate and dissolved to remove the film and expose the multilayer functionalities. This unique strategy resulted in an adhesive system with improved mechanical stability, morphology and optical properties through the inclusion of mussel-inspired molecules, which proved effective against rubbing and ultrasonic treatment.

**Figure 9:**
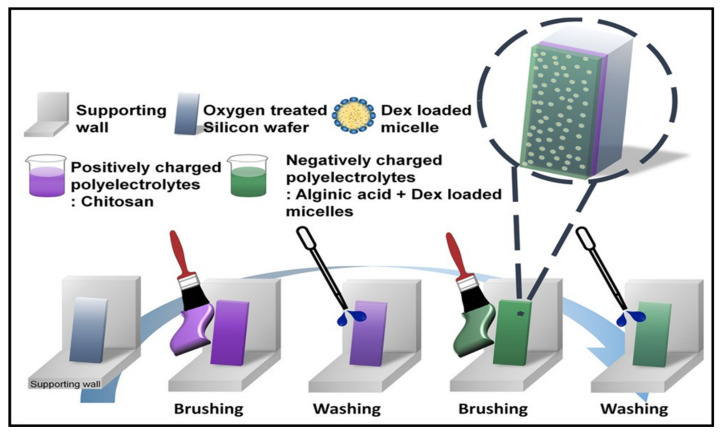
Schematic of a brushed LBL system. Reproduced from [96].

More recently, analogous to the PAT procedure, a rapid and highly efficient LBL assembly technique has been recognised as a process fabricated by a brushed layer. Compared to the conventional LBL method, the preparation of brushed LBL systems is formed through a simple process. The gel assembly involves brushing a PE solution onto a fixed substrate, followed by washing and drying through an air gun and brushing again with an oppositely charged PE, as shown in Figure 9. The steps are then repeated according to the number of bilayers required for the multilayer film required. The advantages of this method were apparent compared to the conventional method, where the technique could expose the typical conventional aspects (uniformity, morphology, thickness, etc.) and attain the ability to fabricate any pattern or shape. In addition, the method could be utilised for drug delivery purposes through site-selective deposition with different film thicknesses [96].

Although this section deals with multilayered gels, some of the gels found in the literature reviewed in this section are perceived as coatings and thin films. However, such structures can be considered as gels when we look at them from a magnified frame of reference. Hence, these examples were chosen to showcase the diversity of PE assembly to form complex structures with the aid of advanced instrumentation. The interdisciplinary approach to PEMs has allowed advanced fabrication methods to develop, where their necessity in applications not only matches the properties exhibited by conventional methods but also displays additional attractiveness such as precision in the structure, size and shape of multilayers at ranging scales. Incorporating PEs as the constituents to create the multilayers further promotes advantages that include arbitrary materials with intrinsic properties that can expand the final PEM tuneability. Despite the growing toolbox in advanced fabrication methods for PEMs, there is a lack of development of assembly in situ to understand and predict how these multilayers can perform based on the properties of the PE and fabrication technique applied.

### 3.2. Tough PE Gels

A material is considered as a tough gel (mechanically strong) if it has the ability to absorb the imposed mechanical energy without fracturing. The major musculoskeletal tissues present in the human body are highly tough and elastic at the same time. For example, a human thoracic spinal disc has a compressive modulus of 15–25 MPa, and articular cartilage has a shear modulus of 0.18–2.5 MPa [97,98]. The development of tough gels which can mimic the mechanical properties of these load-bearing natural tissues is a challenging task in current biomaterial research. The basic principle involved in the design of tough hydrogels is increasing the energy dissipation mechanism in the polymer networks. One efficient way to increase the energy dissipation mechanism in the polymer networks is by introducing reversible physical crosslinks such as polymer chain entanglement, electrostatic interactions, hydrogen bonding, hydrophobic interactions and π–π stacking [99]. In this regard, PE gels provide an excellent platform for facilitating the dissipation mechanism by attaining ionic crosslinks in polycationic and polyanionic polymer networks. Moreover, PE gels produced by ionic crosslinks are relatively stronger and more stable than most physically crosslinked gels. From a molecular perspective, the binding energy of physical crosslinks such as hydrogen bonding is typically around 10 kT [100]. Comparatively, the binding energy arising from ionic crosslinks/interactions is in the range of 8–59 kT [101]. Thus, the difference in these energies at a molecular level significantly impacts the macroscopic properties. For instance, ionically crosslinked ALG/PVA gels using calcium ions are stable and insoluble in water when compared to physical ALG/PVA gels [102]. The ionic crosslinks formed among the α-L-guluronic acid (G) and β-D-mannuronic acid (M) units of ALG and calcium ions are in the form of an egg-shape model, which is highly stable and intact for several weeks [103]. In addition, the long-term stability and mechanical properties of ALG can be tuned by increasing the ALG concentration and varying the cations [104,105]. The increase in the concentration of ALG or divalent ions directly increases the crosslinking density of the PE gel network, thereby directly increasing its modulus/strength.

**Figure 10:**
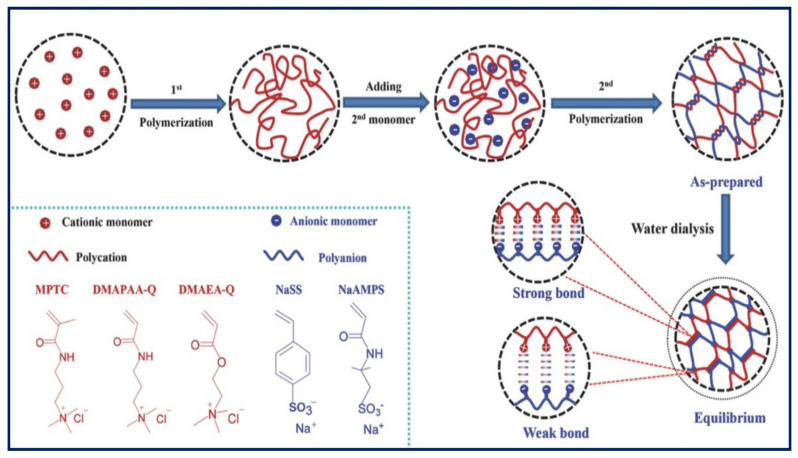
Schematics of the preparation of polyionic hydrogels and the chemical structures of monomers used in this work. After the formation of gel samples, they are dialysed against water to remove the excess counterions and co-ions. As a result, a high density of weak ionic bonds and strong ionic bonds is formed to produce a tough polyionic hydrogel (equilibrium). Reproduced from [107].

In addition to multivalent cations, Komoto et al. [106] employed cationic chitosan produced using sodium hydrogen carbonate for the ionic crosslinking of ALG in the presence of d-gluconolactone (GDL) to produce a PE gel. The resulting PE complex gel demonstrated an elastic modulus of 7000 Pa and was proposed for tissue engineering and scaffold regeneration applications. Employing polycationic and polyanionic (polyionic complexes) is also an efficient way to form a tough PE gel. However, the strong interactions between bulk polycation and polyanion polymers lead to an inhomogeneous precipitation, limiting their fabrication as thin layers produced by layer-by-layer assembly. To overcome this problem, Luo et al. [107] fabricated several tough PE gels by two-step polymerisation between several positively and negatively charged monomers (Figure 10). Briefly, the cationic monomer is homopolymerised and mixed with the anionic monomer and subsequently polymerised with the anionic part. The interactions between the cationic and anionic polymers lead to the formation of a PE gel with both weak and strong ionic bonds. The resulting hydrogels showed a wide range of tuneable mechanical properties, where the combination of cationic acryloyloxethyltrimethylammonium chloride (DMAEA-Q) and anionic sodium p-styrenesulfonate (NaSS) monomers resulted in a very tough gel with a Young modulus of 7.9 MPa.

Constructing interpenetrating networks and double networks, fibre reinforcement, etc., represent efficient ways to improve the toughness of gels. By employing one of the above strategies, Sun et al. [103] constructed an interpenetrating network using ionically crosslinked ALG and covalently crosslinked polyacrylamide, which increase the elastic modulus of ALG from 7 to 29 KPa. In other studies, fibre reinforcement was employed to reinforce an ALG gel; the chemically crosslinked electro-spun gelatin nanofibres introduced into the ionically crosslinked ALG network improved its elastic modulus from ~78 KPa to ~3.21 MPa [108]. Constructing double networks (DNs) is also an important strategy that has shown tremendous improvement in the mechanical strength of several polymer gels [109]. The first DN hydrogel was synthesised by Gong et al. [110] using anionic poly(2-acrylamido-2-methylpropanesulfonic acid) (PAMPS) and neutral poly(acrylamide) (PAAm). Briefly, the first crosslinked network of PAMPS is swollen and inter-spread in the secondary network of PAAm to form a DN gel. Interestingly, the fabricated DN hydrogel demonstrated superior mechanical properties by exhibiting sustainable compressive stress of 17.2 MPa and remained intact even after 93% compression, whereas the PAMPS single-network gel collapsed at less than 50% compression. Since then, the DN structure has received significant attention in the scientific community and has been further extended to natural PE gels to exploit their inherent biocompatibility, which is a prerequisite for biomedical applications. The resulting DN hydrogels have demonstrated a compression modulus of 3.9 MPa and a tensile modulus of 23 MPa, which are significantly higher than their individual counterparts. Similar mechanical reinforcement is observed in other PE gels such as ALG, carrageenan and gellan gum when DN gels are formed using bacterial cellulose.

**Figure 11:**
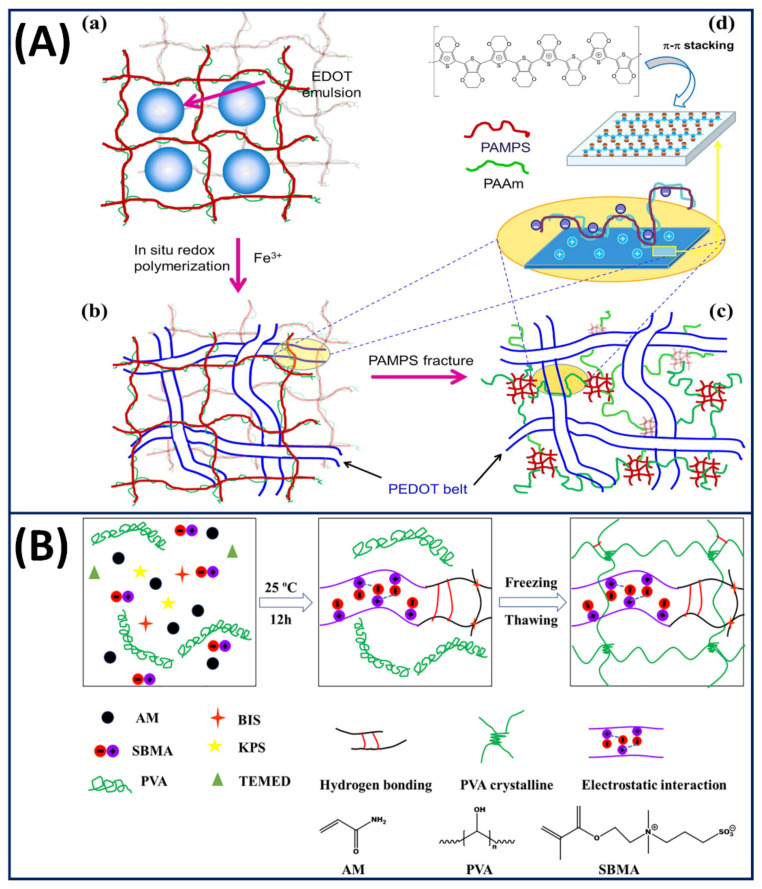
(**A**) Schematic illustration of the synthesis of fatigue-resistant hydrogels with self-assembled PEDOT belts through in situ polymerisation in the double-network matrix. (a,b) A parent PAMPS double-network hydrogel is used to host the emulsion of 3,4-ethylenedioxythiophene (EDOT), which is redox polymerised within the DN gel channels, leading to the formation of an entangled PEDOT belt network. (c) Upon loading, the PAMPS network fractures into fragments linked by the ultralong PAAm chains, yielding a PE network interlacing with the rigid PEDOT belt mesh. (d) The PEDOT belts are composed of self-assemblies of PEDOT chains through π–π stacking. Reprinted (adapted) from [112]. Copyright (2014) American Chemical Society. (**B**) Schematic of fabrication of self-recoverable zwitterionic PVA/P(AM-co-SBMA) DN gels. Reproduced from [32].

There has been significant progress in reinforcing gel mechanical properties by different mechanisms including crosslinking, nanocomposites, fibre reinforcement and double networks. However, most of these tough gels lack self-recovery and are susceptible to cyclic loading (fatigue) because of the damage of irreversible sacrificial bonds during deformation. To overcome this problem, Yin et al. employed polyzwitterions (poly-*N*-(carboxymethyl)-*N*,*N*-dimethyl-2-(methacryloyloxy) ethanaminium (PCDME)) as a building block to construct self-recoverable gels using anionic PAMPS [111]. The self-recovery property on cyclic loading is achieved by simply varying the molar ratio of PCDME and PAMPS, where the reversible sacrificial ionic interactions between PCDME networks are more dominant compared to the irreversible covalent bonds between the PAMPS networks. The self-recovery and fatigue resistance of tough PAMPS/PAAm DN gels can also be improved by introducing poly(3,4-ethylenedioxythiophene) (PEDOT) polymer belts into the double-network matrix by in situ polymerisation of EDOT using Fe^3+^ [112]. During cyclic/repetitive loading, the PEDOT belt interacted with the fractured PAMPS network electrostatically and improved its fatigue resistance (Figure 11A). Moreover, the PEDOT belt in the DN increased the Young modulus from 114 to 335 KPa, and its compressive toughness remained at 1000 J/m^2^ even after several cycles, which is comparable to articular cartilage. Diao et al. [32] proposed a completely new strategy to produce a self-recoverable zwitterionic PE gel by incorporating multiple supramolecular sacrificial bonds into the double network. Briefly, polyvinyl alcohol (PVA) is used as the first network, and the second network comprises covalently crosslinked acrylamide and sulfobetaine methacrylate copolymer (P(AM-co-SBMA)). The hydrogen bonds, crystalline domain and electrostatic interactions between zwitterionic SBMA moieties help dissipate the mechanical energy on loading, and the covalent networks aid in the recovery of the gel structure upon unloading (Figure 11B). A similar strategy was employed by Chen et al. [113] to improve the fatigue resistance of an agar and the copolymerisation of an acrylamide and acrylic acid DN gel by introducing ionic coordination integrations in the second network using Fe^3+^ ions.

Overall, the interactions between polyions and sacrificial ionic bonds in PE gels are successfully employed in the design of tough gels. The rapid reversible crosslinks and the inherent ability to respond to stimuli such as pH or ionic strength render them an interesting candidate for developing fatigue-resistant gels. However, there is significance in developing new design strategies by introducing new supramolecular networks, metal ion complexes and other electrostatic interactions into tough polymer networks for the development of fatigue-resistant tough gels, which are in high demand for load-bearing tissue engineering applications such as spinal disc replacement or knee cartilage replacement.

### 3.3. Bioadhesive PE Gels

Bioadhesives distinguish themselves from other variations of adhesive materials for their inherent ability to adhere to biological surfaces in a biocompatible manner [114]. The adhesion observed can be at a macro- and microscale between the bioadhesive and substrate and between the materials used as well. The interactions between the substrate and the PE gel are initially dictated via the former, where a strong interface is attained via chemical and/or physical interactions. In turn, the covalent or non-covalent bonds formed at the interface will then be subjected to the ability of the PE material itself to remain strong (cohesion) as the interface bond to achieve stability of the bioadhesive whilst adapting to the external environmental conditions [115,116]. The adhesion interactions can thereby be interpreted by the thermodynamic work of adhesion, derived from the Johnson–Kendall–Roberts behaviour, as discussed in previous studies [117,118]. Briefly, one can determine the work of adhesion for bioadhesive PE gels from the relationship between the maximum force required to break a bond (interface or internal) and the size of the gel. In turn, the detachment force obtained will provide information about the strength of adhesion as a function of stress present at the point of maximum detachment.

Despite the vast growth and advancement in the field of bioadhesives, the in vivo application of the materials produced is hindered by their lack of ability to match the nature of the targeted tissue surfaces. For example, some materials impede the diversity in applications by being cytotoxic in nature, while others lack adhesion in wet environments that are considered prerequisites for internal bodily applications such as bioadhesives required for cardiovascular surgery [116,119]. To address such needs, polyelectrolytic gels have demonstrated their ability to incorporate biocompatible materials that lead to strong network interactions whilst also exemplifying their ability to allow a wide tuneability to display attractiveness to diverse applications [120,121,122].

One of the most common methods of obtaining adhesive properties within polyelectrolytic gels is selecting adhesive proteins/polymers as the major constituents that induce adhesion and allow stability in the system. For example, ε-polylysine (EPL) was chosen as the cationic polymer for a polyelectrolytic gel, where the homopolymer could act as an adhesive material by exhibiting strong electrostatic interactions within the system. The homopolyamide showed further attractiveness for selection, where other mechanical properties could be altered and improved such as rheology and temperature sensitivity when in the presence of other materials such as HEP [23]. Comparatively, a hydrogel film was developed with both anionic (HA) and cationic (CHT) layers, which were functionalised with catechol (C) groups to enhance the adhesive properties, where both the cationic and anionic layers exhibited adhesive characteristics. As two adhesive charges were present, C-HA was chosen as the ending layer due to C-CHT being selected as the stable precursor layer for the deposition of the assembly. The study further demonstrated how a higher content of catechol groups improves the wetting contact angle that aids in a stronger bond formation in wet environments, showing potential for tissue engineering [22].

**Figure 12:**
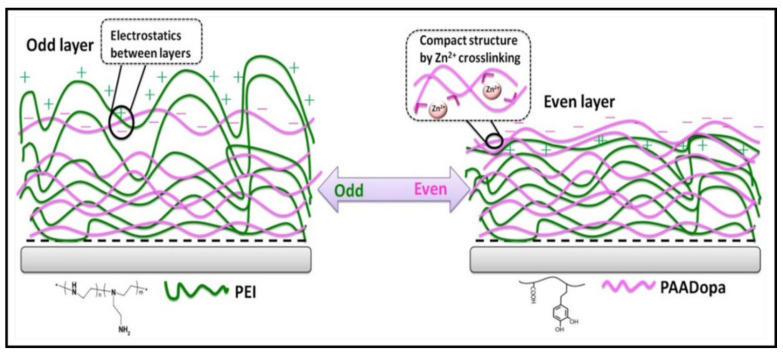
Proposed network structure of odd and even multilayers composed of PAADOPA-Zn and PEI. Reprinted from [126].

More recently, PE bioadhesives have been developed through the mimicry of attractive systems/properties exuded by organisms. Many of these studies involve inspiration gained through organisms found in marine environments, where they exhibit the ability to bind in underwater environments, which is a requirement for biological application within the body. The favourable adhesive properties exuded by mussels (the most common organism researched for underwater adhesion) have been extensively explored in numerous reviews [123,124,125], where 3,4-dihydroxy-phenylalanine (DOPA) moieties are considered the primary source of mussel adhesion in their natural environment. To exploit this property, a DOPA-modified PAA layer crosslinked with zinc was incorporated as the polyanionic component alongside a positively charged PEI layer to determine the effects of LBL assembly of PEs [126]. The multilayered system was comparative to the bioactive C-CHT system. It also demonstrated how obtaining an even number of multilayers within the adhesive component at the topmost layer boosts the overall adhesion capabilities of the polyelectrolytic multilayer [22]. The study demonstrated how the difference in odd- and even-numbered multilayers effects adhesion capabilities not only from their adhesive nature but also their structure, as shown in Figure 12. For instance, an odd-numbered layer with PEI as the outermost layer produced a network dominated by electrostatic interactions with rough surfaces, coils and loops. In contrast, a more compact structure was obtained for an adhesive polyanionic layer dominated by a more crosslinked structure, preventing the absorption of the surrounding liquid environment. Thus, an odd-numbered multilayer presents a more hydrophilic nature with a loosely packed structure, thereby negatively impacting the overall parameters such as thickness, swelling and adhesion [126]. DOPA moieties have further shown their advantages in obtaining excellent underwater adhesion, where Jiao et al. [95] demonstrated how their polyelectrolytic system could withstand ~200 min of ultrasonication with only a negligible change.

Another strategy that can be employed to aid in adhesive characteristics at a cellular level is incorporating natural proteins/polymers, where they are highlighted for their attractive properties for biological application. Advantages are observed in selecting natural materials such as GAGs, where they provide integrin-binding ligands, allowing cell signalling and interactions to occur. Moreover, the addition of other components aids in the tuneability of attractive events such as adhesion, proliferation and cell fate [127]. For instance, HEP-based PEs tend to promote more cell proliferation than chondroitin sulfate, corroborated by the HEP-tanfloc (HEP-TN) PE, where significantly higher cell densities have been observed [128]. Furthermore, the material selection should further be evaluated, as displayed by PEMs fabricated via solely natural polymers, where they exhibited high hydration levels and weak mechanical properties, which can hinder cell adhesion. Thus, integrating other constituents and methods such as post-assembly modification should be assimilated to enable tuneable properties such as adsorption of additional proteins [129]. For example, incorporating ECM proteins (fibronectin and laminin) demonstrated similar results to PEMs formed with CHT and ALG. However, higher-molecular weight proteins are reported to aid cell adhesive and proliferation activities due to their binding abilities [129]. Analogously, synthetic polymers have also demonstrated similar effects. Increasing the molecular weight of PAA was responsible for improving adhesion, resulting in greater interactions from longer PAA chains, providing smaller diffusion coefficients [130].

**Figure 13:**
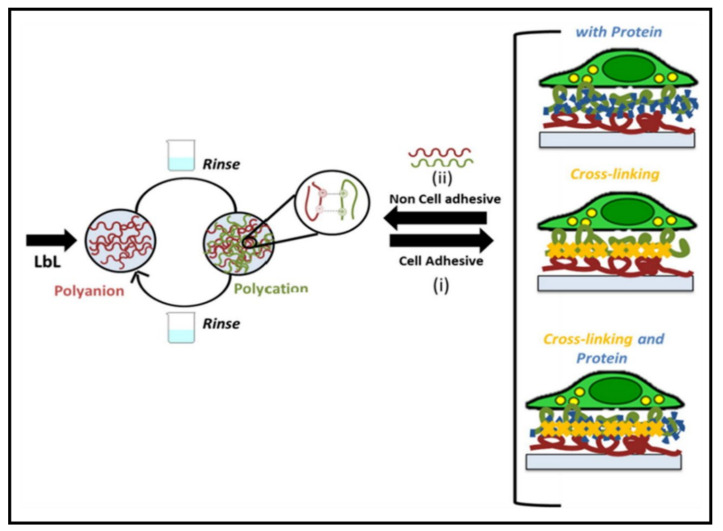
Schematic of PEs displaying electrostatic interactions, becoming either cell-adhesive or non-cell-adhesive based upon the steps used: (i) adhesive properties are gained via the addition of proteins, crosslinking or a combination of both; however, they become (ii) non-cell-adhesive if the cation (CHT) and anion (ALG) multilayers are added on top of the prior mentioned adhesive gaining steps. Reproduced from [129].

An alternative parameter that affects the adhesion characteristics in polyelectrolytic gels is the crosslinked networks formed during the sol–gel transition, as shown in Figure 13. Several studies have demonstrated how the adhesion properties of both polycationic and polyanionic gels are improved through the promotion of interpenetrating networks and chain entanglements between the substrate and surface by the presence of acrylate monomers. For example, Jhiang et al. [131] reported how the co-assembly of poly((trimethylamino)ethyl methacrylate chloride-*co*-sulfobetaine methacrylate) (poly(TMAEMA-*co*-SBMA)) complexed with polyphosphate leads to interactions that result in strong adhesion mechanisms, which include (ii) secondary forces (van der Waals and hydrogen bonding) and (iii) electrostatic interactions through which the common electrical double layer is formed, providing the attractive repetitive sticking ability. Similarly, another study explored the copolymerisation of [2-(Methacryloyloxy) ethyl] dimethyl-(3-sulfopropyl) ammonium hydroxide and 2-acrylamide-2-methylpropanesulfonic acid with the aid of a polyethylene glycol dimethacrylate crosslinker. Not only did the formulation provide strong dipole–dipole interactions between the acrylate moieties, but it further displayed additional non-covalent crosslinking throughout the network. In turn, the ionic monomers could tune the adhesion properties by generating network charges by breaking the electrostatic stoichiometry [122]. Increasing the crosslinker concentration led to numerous findings in a particular study, where it was suggested that it correlated with an increased stiffness, cell number, density and spread area compared to the decrease in the water contact angle and cell circularity [129]. This approach has also been applied for inert compounds, where a wide tuneability was observed in a polyelectrolytic hydrogel via physical crosslinking [23]. Moreover, introducing a secondary crosslinker within systems has displayed the ability to gain mechanical strength, where one study showed an extra boost in the lap shear strength of 30–70% in different environmental conditions through this method [130]. In contrast, a higher degree of crosslinking has also demonstrated stronger interactions within a PE gel that can hinder the functional groups available for binding to tissue surfaces [9]. Thus, a balance in process variables is essential to obtain the targeted application’s desired characteristics.

Comparative to the traditional consensus of attaining oppositely charged components resulting in a PE gel through crosslinking mechanisms, Kim et al. [132] developed the first complexation and coacervation of like-charged PEs inspired by the adhesion mechanism observed in mussels. Unlike many PE complexes (PECs) that exhibit electrostatic interactions, the positively charged PE displayed cation–π interactions, which are deemed stronger in wet environments [121]. This was suggested to result from the thin PE framework with regular-sized pores, compared to the thicker framework coacervates usually have, thus influencing the strong short-ranged cation–π driving forces, enabling a low interfacial energy (<1 mJ/m^2^) in wet environments [132].

**Figure 14:**
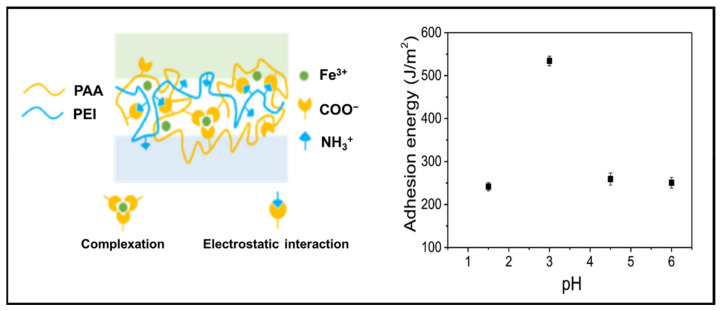
Proposed schematic of interactions of a polyelectrolytic hydrogel and the influence on the adhesion energy based on variations in the pH values of Fe^3+^ (adhesion time of 1 day). Reproduced from [133].

Secondary parameters such as pH, ionic strength and temperature that are associated with the type of constituents used can further impact the adhesion energy and strength. For instance, the adhesion of a hydrogel system consisting of a cationic PEI, anionic PAA and Fe^3+^ displayed a variation in the adhesion energy due to the complexation between Fe^3+^ and PAA due to the change in pH, as seen in Figure 14. The adhesion energy (534 J/m^2^) of the PE complex observed at pH 3 was twice the adhesion energy (~250 J/m^2^) at pH values above and below the optimum condition. This phenomenon was proposed to be due to the chelation between acids and Fe^3+^ ions, which, in turn, would affect their complexation with the carboxyl groups of PAA [133].

On the contrary, Alfhaid et al. [117] demonstrated that raising the ionic strength in the environment results in adhesion loss. To elaborate, the screening of electrostatic charges led to a decrease in the polymeric network, despite adhesion not being a monotonic function of the salt concentration. Moreover, investigation of counterion condensation at different pH levels resulted in different adhesion strengths between both the brush and gel when the constituents were swapped around. The changes were suggested to occur due to the brush’s grafting density, which is associated with thickness, resulting in an increase in the electrostatic energy of the counterions [118]. Comparatively, the presence of weak cations compared to stronger cations was significantly affected by pH conditions. Specifically, a lower pH showed an increase in the multilayer thickness. Thus, a higher surface roughness is more likely to form, which can further be contributed to by high-molecular weight polymers, reducing the overall adhesion strength [128,134]. In comparison to the former systems, a PE hydrogel based on a copolymer with adjacent cation–aromatic sequences was observed to be insensitive to pH due to the presence of quaternary-N, phenyl and functional groups in the system. The hydrogel thereby allowed a great adhesion strength over a wide range of pH, making the system highly attractive for diversity in application [121].

**Figure 15:**
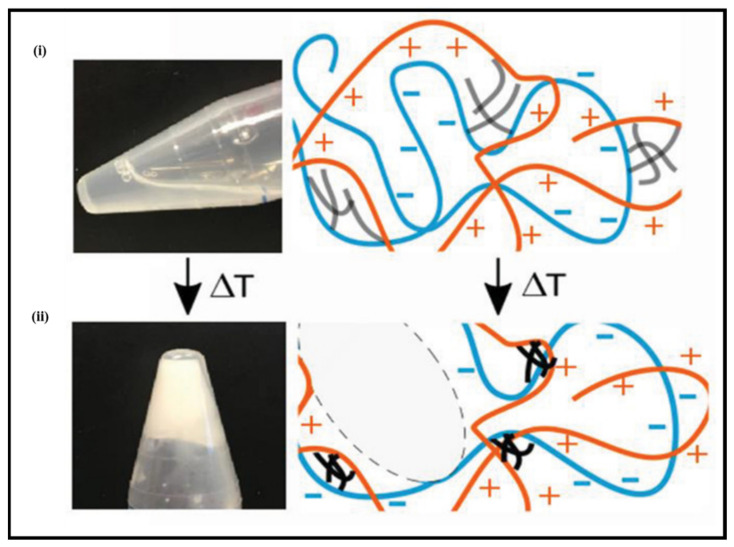
Picture and proposed interactions of the PE complex coacervate (**i**) below and (**ii**) above the LCST of PNIPAM. Reproduced from [120].

Analogously, less common external stimuli have also displayed their potential in tuning the adhesive characteristics of PE gels. One such external stimulus is the temperature, where it has been shown to influence adhesive properties, which tend to correlate linearly with both loss and the storage modulus [23]. For instance, Dompé et al. [120] developed a thermoresponsive complex coacervate PE inspired by the natural mechanisms of sandcastle worms. Copolymer solutions composed of both anionic (PAA) and cationic (poly (dimethylaminopropyl acrylamide) (PDMAPAA)) components grafted onto PNIPAM were mixed, where PNIPAM would aid in the thermoresponsive behaviour of the material (Figure 15). It was demonstrated that an increase in the temperature resulted in an elastic nature observed by rheological data, indicating the formation of physical crosslinks from the PNIPAM chain, leading to a more strengthened material. Although the adhesion strength was not outstanding, it was comparable to other biomimetic underwater adhesives [120]. Correspondingly, the adhesion energy was observed to increase by varying the setting time allowed for bonding. Applying pressure could further increase the bonding strength, yet it was proposed not to have such a significant response as the bonding time [133]. However, these external parameters are dependent on the type of formulation used to develop the PE gel and can differ accordingly.

Embracing the range of factors that can influence bioadhesives’ development, PEs have certainly grown as an attractive material to investigate in the field for future development. PEs allow diversity in the selection of materials whilst also permitting various external and internal parameters to enable the possibility of targeting various tissue types. However, there is a lack of studies demonstrating the in vivo performance of PE bioadhesives and a clear understanding of detailed mechanisms between the adhesive and substrate and within the adhesive itself. Moreover, despite forming strong crosslinked networks, the extent of adhesive capabilities lacks compared to other bioadhesives developed. Thus, additional advancement is required to compete with these standards. Such improvements can be established through the incorporation of more adhesive materials or secondary crosslinks. However, this may hinder other parameters, and therefore a careful balance is required.

### 3.4. 3D Printable PE Gels

**Figure 16:**
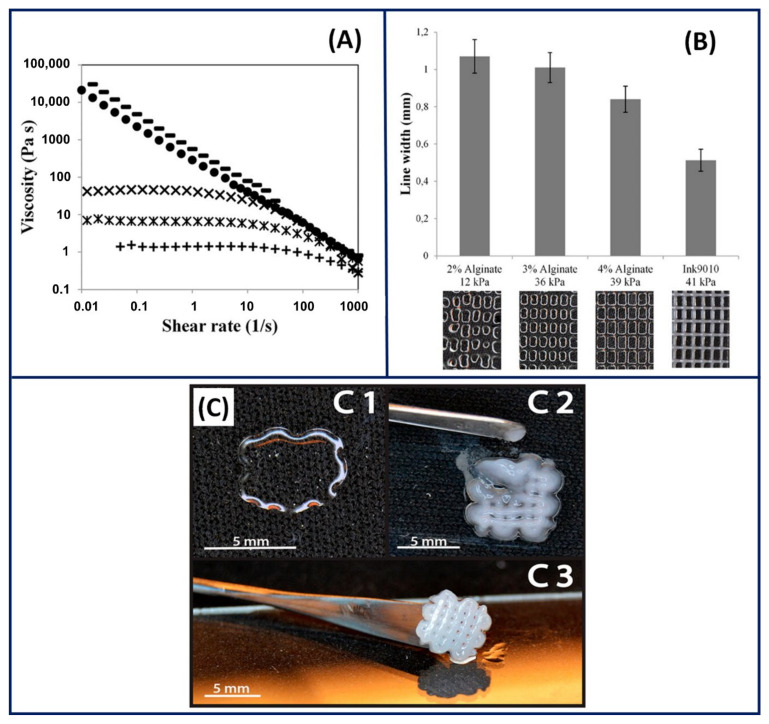
(**A**) Flow curves of 2.5% CNF (−), Ink9010 (•) and ALG solutions: SLG100 4% (×); SLG100 3% (∗) and SLG100 2% (+). (**B**) Line width measurements of 3D-printed large grids with ALG inks compared to Ink9010. The photos below the graph show the printed grids and their different line resolutions. (**C**) Small grid printed with (C1) 3% ALG and (C2) 2.5% CNFs. (C3) Small grid of printed and crosslinked Ink9010. Reprinted (adapted) from [141]. Copyright (2015) American Chemical Society.

3D printing is an emerging fabrication technology that has branched from printing superhard materials to printing soft materials for various biomedical applications [135]. It offers additional advantages over conventional approaches such as design flexibility, customisation and control over physiochemical and biological properties [136]. Although 3D printing has several attractive features, its progress is hampered by the limited choice of materials available. The materials suitable for 3D printing should have prerequisite rheological/flow properties and an immediate solidification mechanism to cope up with the fabrication process [137]. Natural PEs such as ALG have suitable flow characteristics, and their rapid solidification mechanism in the presence of multivalent cations has made them attractive for 3D printing [138]. The favourable flow properties and rapid solidification mechanism of ALG enabled bioprinting of scaffolds by incorporating the desired cell lines for the targeted application. For instance, an ALG solution containing cartilage progenitor cells (CPCs) has been printed in a calcium chloride solution to form a natural vascular system [139]. The solution printed is immediately stabilised by the rapid ionic crosslinking of the ALG solution in the presence of calcium ions. Sithole et al. [140] produced a PE gel complex by printing an ALG/silica solution in a polycationic polymer (polyethyleneimine (PEI)) solution for bone tissue applications. The printed PE complex stabilised by the ionic interactions between ALG and PEI resulted in a robust construct with mechanical properties comparable to bone tissue grafts. However, the printability of ALG by employing calcium ion crosslinking is often compromised due to the concentration limitation posed from the biocompatibility perspective, where the increase in the ALG concentration improves the rheological flow properties but decreases the cell viability. Therefore, several strategies have been explored to manipulate the flow properties of ALG to improve its printability. In this regard, cellulose nanofibres (CNFs) have been used to improve the printability of an ALG gel formed by ionic crosslinking [141]. The addition of CNFs increased the viscosity of ALG and induced the shear thinning mechanism even at low concentrations of ALG (0.25 wt%) (Figure 16A). The line width of the ALG/CNF hybrid (*Ink9010*) is significantly lower than the line width of high-concentration ALG (4 wt%), which directly implies the improvement in its printability (Figure 16B). The increase in the printability of ALG/CNF ink and the rapid sonification characteristics of ALG favoured the formation of a complex yet mechanically stable construct, as shown in Figure 16C. Similarly, graphene oxide (GO) was used by Li et al. [142] to improve the printability of ALG by manipulating the rheological flow properties by exploiting the interactions between ALG and GO. The addition of GO increased the viscosity of ALG and exhibited thixotropic properties, which directly improved the printability. The authors also confirmed that the physical interactions between GO and ALG decreased its spreading and showed shape stability even after 30 s. Furthermore, several studies used external materials to manipulate the flow properties and additional functionalisation of ALG to obtain the desired printability and properties [143,144,145].

**Figure 17:**
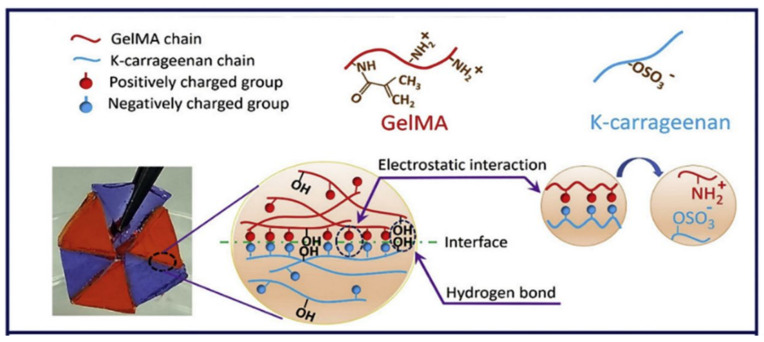
Molecular structure of GelMA and k-carrageenan (Kca). A schematic illustration of the interaction between GelMA and Kca hydrogels. Reprinted (adapted) from [150]. Copyright (2018) American Chemical Society.

Other PEs such as gelatin, CHT and HA have demonstrated their potential for 3D printing for various applications. For instance, a PE gel was fabricated by printing chitosan and subsequently stabilised in sodium hydroxide solution [146]. The resulting chitosan PE gel demonstrated superior swelling and stimuli-responsive bending properties (required for actuators) when compared to the respective casted gel. Gelatin and HA are natural PEs that are widely explored in 3D printing applications. However, in most of the previous studies, both were chemically modified or functionalised to induce favourable rheological flow properties and a rapid sol–gel mechanism by covalent crosslinking [147,148]. Nevertheless, there have been very few studies that investigated the ionic crosslinking of gelatin in combination with other materials to achieve the desired flow properties and printability. For example, Ng et al. [149] fabricated a 3D printable PE gel complex from gelatin and chitosan by exploiting the ionic interactions between them for skin tissue engineering applications. The interactions between the positively charged ammonium ions of chitosan and negatively charged carboxylate groups of ampholytic gelatin improved the overall viscosity, which directly improved the printability. Similarly, the printability of a positively charged gelatin methacrylate (GelMA) gel was improved by combining it with the negatively charged κ-carrageenan (Kca) (Figure 17) [150]. The interactions between the positively and negatively charged polymers improved the printability and mechanical and adhesion strength of the printed construct. Correspondingly, the printability and gelling ability of HA were improved by combining it with ALG, where ALG is used to induce ionic gelation in the presence of calcium ions [151]. The resulting hyaluronic based bio-ink printed in a polylactic acid (PLA) mesh demonstrated suitable characteristics required for articular cartilage regeneration.

**Figure 18:**
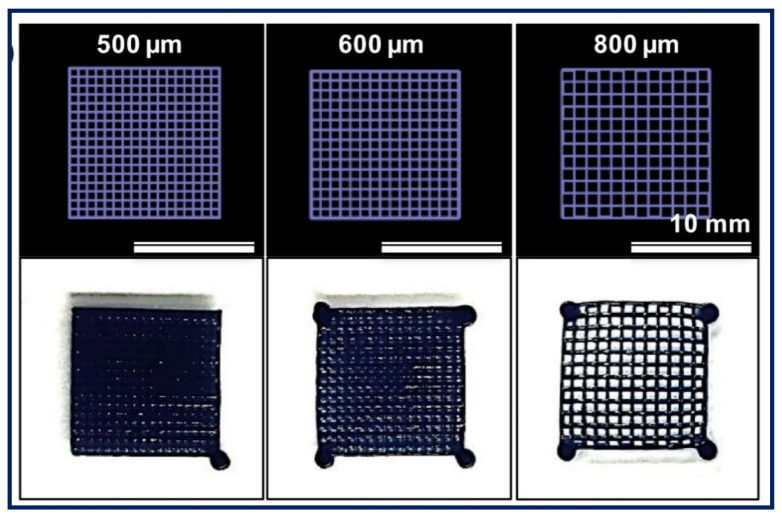
Inputting AutoCAD patterns of parallel squares with different widths (width = 500, 600 and 800 μm) and the resulting patterns using photocurable PEDOT/PSS hydrogels. Reproduced from [152].

Apart from natural PEs, synthetic PEs are also used in 3D printing for various applications such as a 3D printable polyionic complex printed from PEDOT and PSS (Figure 18) stabilised with the aid of photocurable PEGDA for neural tissue engineering applications [152]. The resulting hydrogel demonstrated excellent electrical conductivity and mechanical/structural support, which enhanced neuronal differentiation. Similarly, an ionic composite hydrogel was printed using polyacrylamide (PAAm), 2-(Acryloyloxy)ethyl]trimethylammonium chloride (AETA) and sulfonated silica nanoparticles [153]. The composite hydrogel is stabilised by photocrosslinking the acryl groups in PAAm and AETA, whereas the sulfonate groups on the nanoparticles ionically interact with the quaternary ammonium groups of the photocrosslinked polymer network, resulting in remarkable stiffness, toughness, extensibility and resilience. Very recently, Rahman et al. [154] printed a PE gel using polyvinylidene fluoride (PVDF) and poly(N,N-dimethylacrylamide) (PDMAAm). The preparation of the printable ink involves multiple steps of combining the above polymers and crosslinking agents, followed by stabilising the structure by UV irradiation. The printed construct demonstrated tuneable mechanical, thermal and conductivity properties required for electrochemical device applications.

The number of studies on printable PEs by exploiting ionic interactions is relatively low when compared to their respective modified versions (for example, gelatin can be modified to become GelMA). In the majority of cases, a single polyionic polymer was printed with the aid of external materials, and the printed construct was stabilised using counterions. Moreover, studies exploiting the ionic interactions of 3D-printed PEs did not emphasise enough the importance of those interactions in improving the printability and stability of the construct. One of the main challenges in material design for PE gel 3D printing is control over the rapid sol–gel transition due to strong ionic interactions. Hence, finding a balance between the ionic interactions and the control over the sol–gel transition will be extremely important for the successful printing of PE gels. There has been a significant scope in exploring and improving the printability of polyelectrolytic materials by establishing control over ionic interactions between polyelectrolytic polymers to achieve the desired flow properties and sol–gel kinetics to cope with the printing parameters.

Table 1 show types of PE gels explored in this review for different applications.

**Table 1:** Types of PE gels explored in this review for different applications.

PE Type	PE Gel Composition	Application	Ref
Multilayered gels	PNIPAM-co-MAA nanogel coated with PLL and PGA or CHT and DS	Storage and release of biomolecules	[73]
	PNIPAM-co-AA core and PNIPAM shell microgel coated with PDADMAC and PSS	Drug encapsulation and delivery	[53]
	PNIPAM-co-MAA microgels coated with PLL and PGA or PAH and PAA	Biosensing and controlled release	[75]
	PEI and PSS	Drug encapsulation, microreactors, sensors	[78]
	PMAA nanogels with CS/ALG	Drug encapsulation and delivery	[11]
	CHT/CS on polyurethane discs	Tissue engineering	[84]
	PNIPAM grafted onto CHT/ALG	Drug encapsulation and delivery	[79]
	Polyetherimide and PAA	Plasmonic microstructures and tissue engineering	[95]
Tough gels	ALG/collagen ionically crosslinked using calcium ions	Wound healing	[155]
	CHT and ALG	Drug delivery and tissue engineering	[106]
	DMAEA-Q and NaSS	Fundamental study	[107]
	ALG and polyacrylamide	Fundamental study	[103]
	PAMPS and PAAm	Fundamental study	[110]
	PCDME and PAMPS	Fundamental study	[111]
	PAMPS, PAAm and PEDOT	Load-bearing tissue engineering applications	[112]
	PVA and P(AM-co-SBMA)	Stretchable electronics and energy storage	[32]
Bioadhesive gels	PAA-*g*-PNIPAM and PDMAPAA-*g*-PNIPAM	Tissue engineering	[120]
	P(SBMA-*co*-AMPS) and P(SBMA-*co*-DAC)	Tissue engineering and bioelectronics	[122]
	PVP-PAA and PVA-PAAM gels coated with PEI and PAA	Tissue engineering	[133]
	PMAA and PDMAEMA	Tissue engineering	[118]
	C-HA and C-CHT	Orthopaedic applications	[22]
	DOPA-PAA and PEI crosslinked with zinc	Drug delivery, biosensing, tissue engineering	[126]
	Poly(TMAEMA-*co*-SBMA) and polyphosphate	Tissue engineering, drug delivery and biosensing	[131]
	Chitooligosaccharide and poly(*N*-acryloyl 2-glycine)	Tissue engineering	[9]
	PEI and PAA complexed with iron ions	Tissue engineering	[133]
3D printable gels	ALG and calcium ions	Tissue engineering	[139]
	ALG and PEI	Bone tissue engineering	[140]
	ALG and acrylamide	Fundamental study	[144]
	Chitosan and sodium hydroxide	Soft actuator	[146]
	Gelatin and chitosan	Skin tissue engineering	[149]
	GelMA and κ-carrageenan	Tissue engineering	[150]
	HA and ALG	Tissue engineering	[151]
	PEDOT: PSS and PEGDA	Neural tissue engineering	[152]
	PAAm, AETA and silica nanoparticles	Sensors and robotics	[153]
	PVDF and PDMAAm	Electrochemical devices	[154]

## 4. Applications

With the combination of the attractive characteristics and different types of PE gels explored, the potential to address a range of applications is highly foreseeable; the primary reasons being that PE gels can utilise biocompatible materials that have shown their ability to respond to physical (temperature, light, etc.), chemical (pH, salt, etc.) and mechanical (forces, displacements, etc.) variables. Therefore, a notable appeal in PE gels is particularly drawn towards those that align with biomedical applications. Such applications in the biomedical field include PE gels used as actuators, electronics, microfluidic chips, drug delivery systems, cell cultures and tissue engineering materials [156,157,158,159,160,161]. Within the scope of this review, the authors will cover a few biomedical applications in which PE gels have shown a current promising impact in their respective field.

### 4.1. Tissue Engineering

Tissue engineering embraces strategies that focus on creating a platform for new biological tissue through regeneration, maintenance or replacement of damaged structures. In this regard, hydrogels have displayed high attraction in the field, where they can provide strong mechanical support for cells and tissues, which enables cell proliferation and survival by mimicking the native target tissue [162,163]. Although conventional hydrogels can attain a range in shape flexibility and swelling to relieve weakened target tissues, there is still much focus on tuning the durability, mechanical and swelling properties to allow variation for specific applications. A promising approach to address this is PE gels, where they can be modulated in a facile manner through ionic functionalization, allowing enhancement in mechanical stability, durability and degradability [164]. Moreover, as the constituents used to develop PE gels typically attain properties originating from biological tissue [22,23], they can match biological motions at the molecular level from their stimuli-responsive behaviour [165]. Thus, PE gels are gaining further attention as a candidate for tissue engineering applications, where they can address specific properties required by the targeted tissue.

With the ability of some organisms to display self-healing properties, researchers were inspired to mimic this unique characteristic to develop self-healing hydrogels for biological tissue [166,167,168]. Self-healing hydrogels are generally defined by physical or chemical processes at the molecular level. A few studies have shown this attractive self-healing behaviour through the former process. For instance, a PE gel composed of CHT and fibrin displayed self-healing properties via interpenetrating structures formed through Schiff–base crosslinkages, allowing injection of the gel and enabling recovery of the blood circulation in the hindlimbs of mice [169]. However, chemical crosslinks are generally limited in application due to the strong bonds formed during gel preparation, which is not a constraint for physical crosslinks [170]. Despite this slight advantage, physical gels typically exhibit low strain, resulting in the possibility of network failure [171].

**Figure 19:**
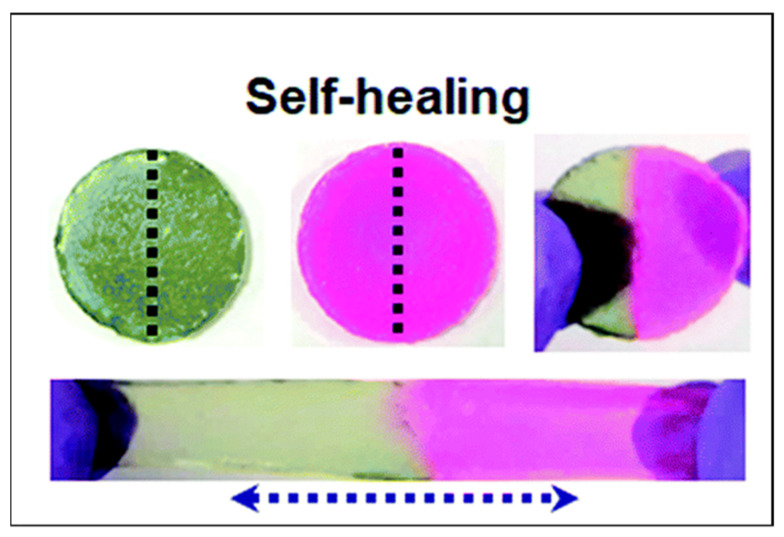
Image of self-healing PE gels, with gels cut (dashed line) and self-healed represented by the dye and original gel. Reproduced from [173].

On the other hand, supramolecular hydrogels stand out in that they achieve self-healing properties and entail tough properties [172]. Physical hydrogels that attain these properties can be prepared by PEs, where they typically display ionic mechanisms between oppositely charged ions [161,173]. However, to circumvent the strong associations of the strong interactions formed, polymerisation techniques are an attractive method to overcome this obstacle. To attain both toughness and self-healing properties, a polymerisation technique was utilised, where PEC coacervate gels were formed by mixing anionic polyacid microgels and cationic PEI [173]. Self-healing was attributed to the PEI chains that interpenetrate the anionic microgel, allowing for greater ionic crosslinks, where self-healing efficiency was reported at 92%. The study also demonstrated that the coacervate gel could be toughened by adding Ca^2+^ through further ionic crosslinking. In turn, the PE gel could be cut and joined back together whilst maintaining the tough strength, as shown in Figure 19. In turn, attaining both toughness and self-healing properties allows PE gels to be an attractive material for targeting applications such as wound healing and potentially cartilage repair.

Strategies employed to repair and maintain some tissues such as articular cartilage are quite difficult, where they have a limited calibre for self-healing as a result of the limited blood supply [163]. Notwithstanding, conventional hydrogels having the ability to act as strong scaffolds are deemed inefficient for cartilage tissue applications as they are hindered through their weak mechanical properties [109]. Conversely, PE gels can attain a high fracture stress (8 MPa) and fracture energy (4000 J/m^2^), that exceed conventional hydrogels (~0.1 MPa and ~10 J/m^2^), due to DNs [174] formation, and match the compressive stress of articular cartilage [109]. For instance, Fan et al. [175] developed a DN hydrogel that resulted in a fracture stress value of 8.38 ± 0.67 MPa, comparable to articular cartilage tissue. The gain in mechanical strength was attributed to the DN’s polymer content and mass ratio, which contributed to the swelling properties of the PE network. Furthermore, the interpenetrated network also enhanced the mechanical strength to allow stress transfer, thereby avoiding stress concentrations. In an analogous study, DN PE-based gels were developed to be not only tough but also highly stretchable [103]. The gel could be stretched over 20 times its initial length with fracture energies of ~9000 J/m^2^ due to the synergy of the hysteresis of the ionic network and crack bridging of the covalent crosslinks. In turn, the PE gel displayed potential for articular cartilage application.

More recently, considerable research has been performed on stimuli-responsive natures, where PEs have displayed a wide range of hydrogels for tissue engineering applications. These hydrogels are more commonly noted as “smart hydrogels” due to their ability to respond to changes in external stimuli [163,165,176]. Based on the type of stimulus, PE gels can be physically or chemically responsive, where the latter is more commonly used for the exploitation of ionic environments [128,133,134], such as DN PE gels. Physically responsive PEs have gained attractiveness, particularly for injectable applications. For instance, as PNIPAM exhibits a thermoresponsive nature, axionically charged PAA and catatonically charged PDMAPAA were separately grafted onto PNIPAM, followed by mixing the grafted copolymer solutions. The resulting PE coacervate gel could then be thermally activated through a phase transition and be utilised for injectable applications [120]. Analogously, physically responsive PEs have also been used for unique applications such as neural tissue engineering, where electrical cues can be used to promote neurite outgrowth. This phenomenon was demonstrated by a PE composed of the sodium salt of poly(γ-benzyl-L-glutamate)-r-poly(L-glutamic acid) (PBGA20-Na), where the exploitation of the electrical stimulus was capable due to the inclusion of the neurotransmitter glutamic acid within the PEC. Moreover, it was suggested that the sodium ions further aid in action potentials, influencing the neurite growth from the electrical stimulus [177]. These few examples demonstrate how attaining a smart feature within a PE system is desirable, where it can mimic the native responsiveness of healthy tissue, enabling it to self-regulate and function.

From these few examples explored, PE gels have demonstrated high potential in tissue engineering application, whether it be through self-healing, mechanical strength or smart responsive behaviour. For these gels to be more resourceful, the regenerative performance should foremost be enhanced, where improvements can be made in promoting cell spreading, proliferation and differentiation. On another note, for the gels that have been studied, there have been limited applications tested on animals, where the viability and performance in the human body are still open for investigation.

### 4.2. Drug Delivery

In recent years, PEs have been vastly explored for pharmaceutical applications, where significant growth is observed in the development of PECs for drug delivery systems (DDSs). For a material to be deemed applicable for a DDS, it must meet the fundamental criteria of attaining biocompatibility, biodegradability (controlled degradation rate), cytocompatibility and complex stability of the materials. In turn, the selection of the material will further influence other properties such as the morphology, adhesion, drug loading and release profile, which will need to be controlled accordingly. Favourable attractiveness is observed in utilising PEs for DDSs, where they not only reach the aforementioned criteria but also present ease of preparation and incorporation of bioactive compounds, avoid hindering cell function, display the use of water as a solvent during preparation (benefiting feature for DDSs for human applications) and are typically produced as hydrogels in situ, which closely match the properties of biological tissue, thus enabling a safe route for in vivo applications [178,179,180].

PEs selected for DDSs are broadly developed in the form of nanoparticles (NPs). These NPs exhibit their advantages over conventional DDSs as they present themselves in submicron size, enhancing the system’s stability through an extended molecule half-life whilst also producing a high surface area, hence leading to greater absorption properties in contrast to DDSs utilising larger carriers [181].

**Figure 20:**
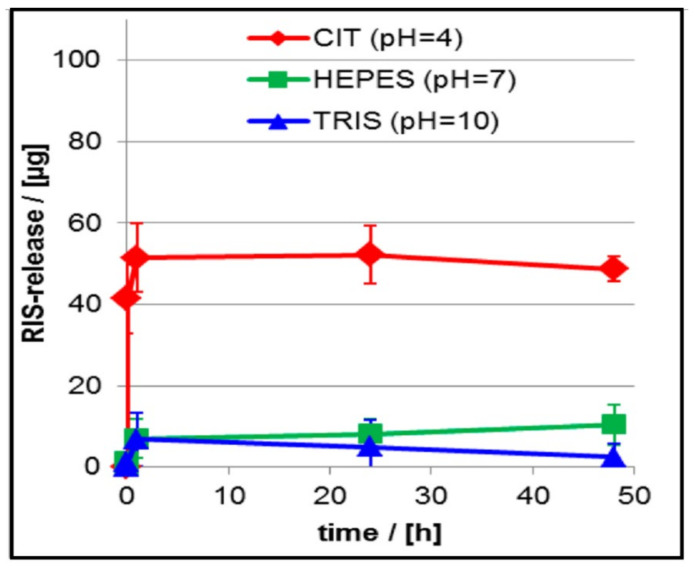
Time-dependent release of the drug risedronate from a DDS based on cationic PLL and anionic cellulose sulfate (CS) placed in aqueous release media of citric acid monohydrate at pH 4, 2-(4-(2-Hydroxyethyl)-1-piperazinyl)-ethane sulfonic acid at pH 7 and tris(hydroxymethyl)-aminomethane at pH 10. Reproduced from [179].

Natural-based polysaccharides employed as NPs for DDSs are not only useful as carriers but are also beneficial as they exhibit physicochemical and pharmaceutical properties such as bacterial uptake, solubility and permeability [182]. The most common natural polysaccharide used is CHT due to its cationic nature, mucoadhesive properties and ability to act as a permeation enhancer, thereby allowing facile construction of PEC NPs at mild conditions [183]. This natural polysaccharide has also been used as a drug barrier, enabling a controlled release of the drug [10]. Additional natural polysaccharides have also been incorporated to form PECs such as dextran, sulfate, cellulose and ALG, where some have even been complexed with CHT to exploit combinatory properties [179,184,185]. Kilicarslan et al. [185] developed a PEC film composed of ALG and CHT for the application of periodontal therapy as a DDS. The complexation ratio of higher concentrations of ALG and low molecular weights of CHT aided in the slow-release kinetics of the drug. However, a limitation met with using CHT for DDSs is that it generally requires acidic additives to aid in solubilisation. Therefore, trimethylchitosan (TMC), a derivative of CHT, is utilised as a substitute, where it attains the natural properties of CHT in addition to a permeation advancement and solubility over a wider range of pH [186]. Sequentially, it simplifies the method whilst eliminating the possibility of acid-catalysed degradation, thus withholding bioactive properties in the DDS. To exemplify the advantages, the development of a PEC made of the CHT derivate and HEP resulted in a release of 85–90% of the drug in a pH medium of 6.8 and 7.4, although a significantly lower drug release was displayed at a pH of 1.2, therefore indicating that, despite having the ability to release the drug over a wide pH, the medium does influence the release of the drug [159]. It could be inferred that when there is a change in pH values in the release medium compared to the pH value at preparation, the amount of the drug charges can decrease with decreasing pH values, thus resulting in a higher release of the content of the drug [179], as displayed in Figure 20.

**Figure 21:**
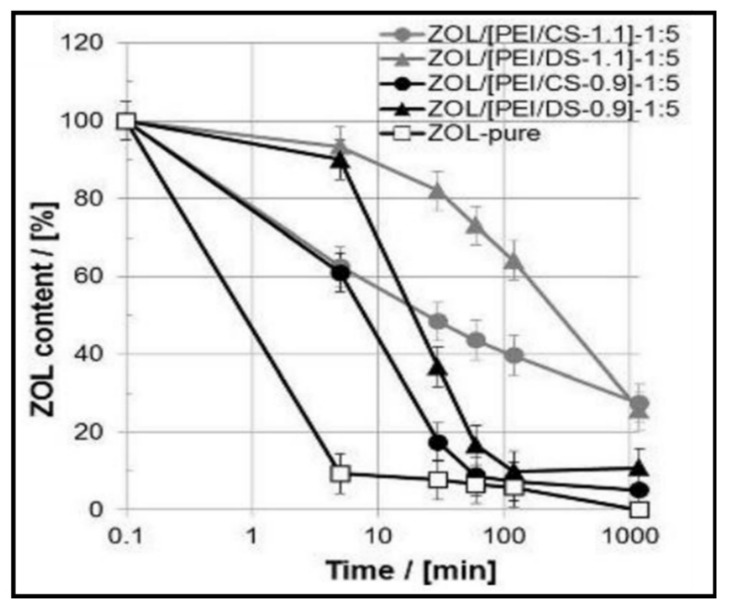
Release kinetics of PEI/CS and PEI/DS PE films based on different mixing ratios of the cation and anion content. Reproduced from [184].

Apart from polysaccharides, DDSs have also incorporated natural- or synthetic-based polymers such as PEI, poly(_L_-lysine), poly(_L_-glutamic acid) and PAA [179,187,188]. In an overview of different PEC combinations, it was demonstrated how the selection of the polymer and the construction of the system can affect several properties of the DDS [189]. Wet adhesion was only visible in ternary systems composed of the drug zoledronate (ZOL), PEI and CS, compared to binary systems composed of this drug and the two oppositely charged constituents. This was proposed to occur due to the stronger adhesive and cohesive forces that rendered particles tougher, leading to further entanglement and stabilisation. The combination of PEI and CS was further shown to improve the cytocompatibility and drug retention of the DDS. Both factors were due to the DDS displaying a branched polycation/linear polyanion combination in comparison to a branched/branched system; where the former would (i) avoid steric effects and block the charged toxic amino groups of PEI-thereby matching tissue culture [190], and (ii) display structural compactness due to the strong electrostatic binding with the cationic PEI- allowing higher drug retention [189]. The PEC NP outer and inner shell structure has also been shown to affect drug retention, displayed by PEI/CS and PEI/DS PE films (Figure 21). As an anionic drug (such as ZOL) encounters negative charges on the outer shell of a PEC with a mixing ratio of Q = 1.1 (anionic system), this leads to electrostatic repulsion. In comparison, for a PEC with Q = 0.9 (cationic system), the positively charged outer shell accelerates the anionic drug release from the inside of the PEC by electrostatic attractions [184].

**Figure 22:**
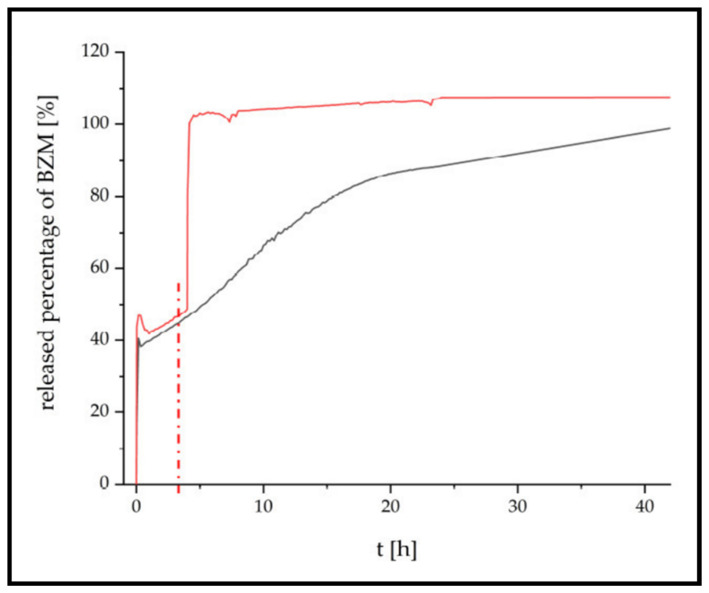
Release kinetics of bortezomib in a DDS composed of anionic poly(caffeic acid) and polycationic PNIPAM-co-dimethylaminoethylmethacrylate, where the temperature is increased from room temperature (black) to 42 °C (dashed line) after four hours. Reprinted from [191].

Variations in swelling and thermoresponsive properties have further been shown to demonstrate an influence on drug release kinetics, such as an in situ gelling PEC composed of CHT, gellan gum and ondansetron hydrochloride (drug). The PEC demonstrated how the formulation altered the swelling and drug release capabilities. Due to the swelling present in the CHT/gellan gum complex, a higher water uptake could be observed, resulting in a slow drug release with negligible burst release. This could be further explained as the swelling led to a viscous gel layer on the matrix’s outermost layer, thus leading to slower release kinetics of the drug [180]. Similarly, the concentration, volume and molecular weight of certain constituents used could alter the complexation of the PEC, thereby resulting in either an increase or decrease in the drug release profile [185]. Swelling properties have also been seen to increase the PEC particle size when exposed to an ionic environment, thereby increasing the loading capacity of the drug [187]. On the other hand, attaining a thermoresponsive system allows the release of the drug in a controlled manner, reducing the possibility of burst release and control of the concentration decrease in the properties required. Figure 22 illustrates a study, where increasing the temperature stimulus from room temperature to 42 °C accelerated the elution of the drug due to the changes in conformation, leading to easier access [191]. Therefore, it would be a more attractive concept where the elution of the drug is thermoresponsive to the PEs, allowing a spatiotemporal release [189].

Another major influence that favours the performance of PEC DDSs is the ability to tune mechanical properties. Interactions that occur within the PEC are observed to influence the range of tuneability of the DDS directly. For example, a polyelectrolytic–drug complex was easily formed through ionic interactions and loading of the drug ciprofloxacin (CIP), where different dispersions led to mechanical property changes, which could further be tuned by modifying the amount of crosslinking points available in the polyelectrolytic network. Therefore, the mechanical properties could be elevated if strong ionic interactions could form between the amino groups of CIP and the acid groups of the PEC, resulting in the hardness of the overall polymeric network [182]. Correspondingly, a PEC film’s mechanical rigidity was altered through the addition of the drug tobramycin. The increase in mechanical rigidity was due to a co-nonsolvency effect, further resulting in increased adhesion. The drug was additionally seen to compete with water molecules and PAA chains, leading to further structural changes [188]. Thus, tuning the drug’s concentration in correlation with the constituents in the PEC and the surrounding medium could enhance the mechanical properties of the system.

Optimisation of PECs has resulted in enhanced DDSs, mainly associated with the inclusion of a centrifugation step. Centrifugation enables the system to remove the supernatant present, where the pellet phase (coacervate) can be separated and redispersed in freshwater again, thereby creating a more colloid stable system. Furthermore, centrifugation of the system displays the ability of the bound PEs to be constant at a varying molar concentration range of anionic to cationic species, which validates the adhesive stability in comparison to an uncentrifuged system that is not stable. Therefore, it can be implied that the centrifugation process removes any excess PEs regardless of the mixing ratio to produce a stabilised coating [179,187].

PECs have demonstrated interesting properties that stand out as an attractive option for DDSs. They have shown their ability to attain stable complexes with wet adhesion, cell compatibility, biodegradability and controlled release [179,185,190]. Having a stable complexed system for a long duration (weeks–months) further allows ease of transport and application in the medical industry. Therefore, utilising PECs provides an adaptable, tuneable, compatible and simplistic approach for various types of delivery systems of therapeutic drugs, although there are obstacles that need addressing in relation to the internal behaviour of PEs in cells, where information in vivo is limited. However, it is heading in a promising direction, where the pharmacokinetics of a drug and carrier composed of a PEM-based DDS have been explored [192].

### 4.3. Actuators

**Figure 23:**
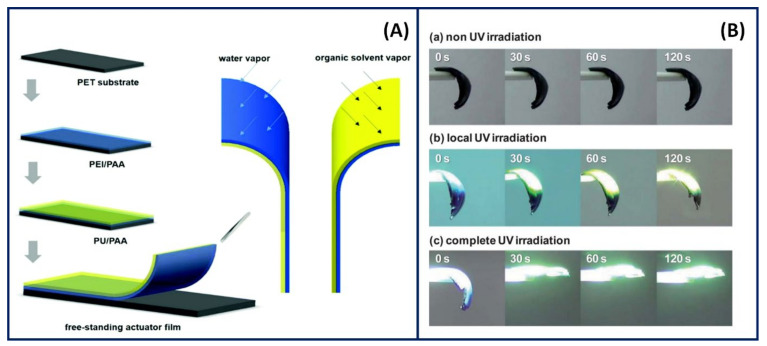
(**A**) Schematic presentation of the preparation of free-standing (PEI/PAA)/(PU/PAA) multilayer actuator membranes, which are responsive to water and organic solvent vapours. Reproduced from [156]. (**B**) Photographs of 1.0 mm-thick NBA and bromocresol green-loaded palm-shaped bilayer gels consisting of polyacid P(NIPAAm-co-CIPAAm) and polybase P(NIPAAm-co-DMAPAAm) layers in a time sequence under the illumination of a UV lamp on the polyacid side of the gels (c). As a control, gels were exposed to ambient light (a). To demonstrate local UV exposure, one half of the bilayer gel was continuously exposed to UV light (b). The colour of the gel started to change instantaneously from blue to yellow (pH < 3.8) in the photo-illuminated region. Reproduced from [196].

With increasing growth in technology and the implementation of automation and robotics in our daily lives, actuators are visualised as an attractive option to adopt, especially for biomedical applications [193]. The materials that can exhibit mechanical motion when subjected to external stimuli (temperature, pressure, light, electricity, magnetic) are promising candidates for developing actuators [194]. In this regard, PE gels are excellent materials because of their ability to respond to external stimuli due to the ionisable groups present in the polymer backbone. For instance, a PE gel actuator was produced by exploiting the interfacial interactions of oppositely charged polymers such as 2-acrylamido-2-methyl-propanesulfonic acid (AMPS—negatively charged) and dimethyl aminoethyl methacrylate methyl chloride (DMC—positively charged) monomers [16]. The fabricated PE gel actuator showed attractive swelling properties and rapid actuation (bending) in salt solutions under an electric field. The bending speed or actuation of the PE gel can be controlled by the strength of the electric field applied and the charge density present in the gels. A salt-responsive actuator was designed by constructing bilayers using polycationic poly([2-(methacryloyloxy)ethyl]trimethylammonium chloride/N-(2-hydroxyethyl) acrylamide (polyMETAC/HEAA) and polyzwitterionic poly(3-(1-(4-vinylbenzyl)-1H-benzo[d]imidazol-3-ium-3-yl)propane-1-sulfonate) (polyVBIPS) layers [195]. Both the layers exhibited salt-responsive swelling and shrinking properties in opposite directions, thereby allowing the actuator to perform maximum bending. Comparatively, multilayer self-assembly of PE thin films showed ultra-fast and large actuation [156]. A multilayer film produced by LBL assembly of PEI/PAA and polyurethane (PU)/PAA PEs exhibited different swelling and shrinking properties in water and other organic solvents, providing the actuation property (Figure 23A). The fabricated actuator showed swelling and bending properties when exposed to external environments and controlled actuation by altering the thickness ratio of the two layers. The excellent responsive characteristics of PEs are also exploited in developing photo-sensitive actuators. A photo-switchable, pH-responsive bilayer actuator was fabricated by combining oppositely charged poly(*N*-isopropylacrylamide-co-2-carboxyisopropylacrylamide) (P(NIPAAm-co-CIPAAm)) and poly(*N*-isopropylacrylamide-co-*N*,*N*′-dimethylaminopropylacylamide) (P(NIPAAm-co-DMAPAAm)) layers [196]. The fabricated bilayer hydrogel showed quick photon release when subjected to UV irradiation, thereby triggering the pH change which allows the gel actuator to bend rapidly (Figure 23B).

### 4.4. Bioelectronics

With the advancement in electronics, mimicry of mechanical properties of biological tissue has enabled the rise in bioelectronics. Therefore, bioelectronics is regarded as a discipline between the convergence of electronics and biological systems [197]. Amongst all the properties exhibited by PEs, ionic conductivity is considered the principal factor required for bioelectronics. The reason is that the ionic mobility and charge carriers present within PEs considerably influence the electronic device’s final sensitivity and conductivity [198]. Thus, PEs present themselves as a prospective option, where various strategies can be employed to develop bioelectronics.

**Figure 24:**
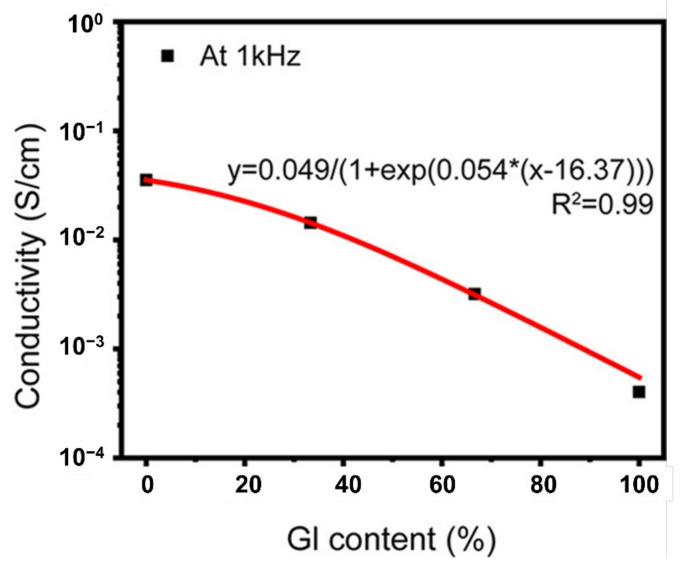
Conductivity of PE water–glycerol gel, with varying glycerol contents. Reprinted (adapted) with permission from [157]. Copyright (2019) American Chemical Society.

The development of PE gels for bioelectronics has emerged through simplistic techniques that address next-generation designs for biodevices. For instance, hydrogels composed of oppositely charged gelatin (cation) and chondroitin sulfate (anion) were simply complexed through electrostatic interactions to form PECs and then centrifuged to allow a hydrogel-like material. Bioelectronic attractiveness was demonstrated from the study, where the PE gel could infiltrate the porous structure of carbon electrodes to enhance the charge transfer at the electrolyte and electrode interphase whilst retaining the polymeric network structure [199]. In an analogous study, PE gels with added glycerol were prepared by radical polymerisation. Increasing the glycerol content resulted in a loss in conductivity, which suggested it decreased the free ions present in the hydrogel (Figure 24). Thus, by controlling the content of glycerol in the hydrogel, the required conductive properties could be attained for various applications [157].

Another unique approach for bioelectronics entails using conjugated PEs (CPEs) which are known as a class of organic materials, where the charged electrolyte groups are covalently bound to a conjugated backbone. CPEs can then be subdivided into two categories (cationic or anionic) based upon their net charge in the side groups [15]. Within these categories, structural differences in conjugated backbones are suggested to result in different conducting states. For instance, poly(3-thienyl)ethoxybutanesulfonate exists in a semi-conductive state. Semi-conductive states form when the pendant ions are simply compensated via the counterions present. In contrast, poly(3,4-ethylenedioxythiophene)sulfonate (PEDOTS) is highly conductive. The conducting state occurs for PEDOTS as the lone pairs of two oxygens can stabilise the positive charges from the backbone via electron donation [200]. Exploiting this feature, Persson et al. [201] demonstrated how a derivative of PEDOTS (with higher self-doping) resulted in a homogeneous electronic material with the capability of controlling cell detachment, due to oxidation states, where the material further improved the preservation of cell surface antigens.

Similarly, other thiophene-based CPEs doped with anions allowed the function of an organic electrochemical transistor (OECT) via a liquid medium composed of ethylene glycol (EG). The CPE was suggested to have high potential as an OECT as the combination of EG and CPE improved the hole anion transport and temporal response attributed to an ordered morphology and faster transport of ions [202]. Expanding the potential of OECTs, Pappa et al. [14] added to PEMs through LBL assembly composed of PLL and PSS on top of a PEDOT/PSS OECT channel. The addition of the PEMs allowed versatile tuning abilities such as thickness, roughness, softness and charge that could further modulate the electrical potential of the OECT. Further tuneability has been exhibited by conjugated PE blends that have been shown to improve oxidation stability in addition to optical and electrical parameters, thereby showing promise as an attractive material for OECTs [203].

Another type of bioelectronics device investigated is PE gel diodes (PGDs). As most electronic materials have a poor combination of flexibility, conductivity and biocompatibility, PGDs stand out as promising materials that address these requirements whilst also being cheap. PGDs are composed of oppositely charged backbones that make contact, resulting in an interface that consists of a counterion depletion layer. This interface allows for a rectification of the current, which is an attractive property for ionically driven devices such as biointegrable electronics [204,205]. Experimentally, studies have demonstrated higher salt concentrations hinder the rectification of charges, where the electrostatic potentials further influence the behaviour of a PGD, thus suggesting the performance of a PGD is dependent on the native interactions found in the counterions and backbone of the gel [206,207]. However, the current advancement in PGDs is more revolved around theoretical studies and quantitative calculations. A prominent model that first explored these studies demonstrated the primary influences of PGDs to be electrochemical reactions at the interface and the voltage applied, which allows predictions of the rectifying behaviour via potential drops [208]. More recent studies have attempted to broaden the diversity in the applicability of the model for molecular simulations. However, the model’s validity has been questionable with the methodology utilised [50,209].

With the ever-growing bioelectronics field, PE gels have shown their capability to meet the demands met with current trends. A major factor influencing the use of PEs in comparison to other materials arises from the properties they exhibit, such as biocompatibility, conductiveness and cost-effectiveness [210,211,212] in an application. However, major areas need much growth to make a large impact, such as PGDs and OECTs, where advanced manipulation of PEs is yet to be thoroughly explored. To stand out further, an exploration into in vivo bioelectronics will aid PE gels to protrude as an option, which is an area with limited studies.

## 5. Conclusions and Future Recommendations

In summary, this review embraces the recent research on the fabrication of various types of PE gels with ionic and crosslinking mechanisms, with a specific emphasis on highlighting their stimuli-responsive properties. The fabrication of multilayer PE gels using advanced approaches (DPN, PAT) allows the construction of complex patterns with a high resolution at the nanoscale. PE gels have also shown excellent tuneability of toughness and mechanical properties in order to dissipate the mechanical energy imposed. However, there is significant scope in developing fatigue-resistant tough PE gels by introducing supramolecular networks, metal ion complexes and other electrostatic interactions into the tough polymer networks. On the other hand, PE bioadhesive gels have demonstrated various strategies to gain adhesion strength, stimuli responsiveness and other desirable characters. PEs have also been explored in developing printable hydrogels, where a majority of the studies used a modified version of a PE or employed counterions to stabilise the PE gel after immediate printing. There have been very few studies on 3D printing of both polycationic and polyanionic polymers simultaneously because of complexities posed from the rheological flow perspective and limited control over the sol–gel kinetics.

Overall, there has been significant progress in constructing multilayer PE gels by exploiting the ionic interactions. However, there has been relatively less progress in tough, adhesive and printable PE gels when compared to conventional hydrogels’ progress because of the complexities involved in understanding the dynamic nature of ionic polymer networks. Nevertheless, a clear understanding of the complexities posed by the ionic networks in PE gels coupled with their attractive stimuli-responsive nature and advanced fabrication devices can extend the scope of PE gels’ utility for biomedical and industrial applications.

The emerging biomedical applications of PE gels are based on exploiting PEs in the form of nano- and microgels, which show promise in significantly improving the mechanical properties of PE gels. Similarly, with the rapidly emerging field of digitisation and electronics, research should focus on studies of PE gels transitioning from 3D- to 4D-printed structures that evolve as a function of time or other stimuli in a predictable way.

## Data Availability

This is a review article and the study did not report any new data.
